# Inverse Modeling Identifies Efficient Emission Control Strategy for Mitigating PM_2.5_ Pollution

**DOI:** 10.1021/acs.est.6c06581

**Published:** 2026-09-29

**Authors:** Chirag Manchanda, Libby H. Koolik, Alper Ünal, Inez Fung, Julian D. Marshall, Rachel Morello-Frosch, Alexander J. Turner, Robert A. Harley, Joshua S. Apte

**Affiliations:** † Department of Civil and Environmental Engineering, 1438 University of California, Berkeley, California 94720, United States; ‡ Eurasia Institute of Earth Sciences, Istanbul Technical University, Istanbul 34469, Turkey; § Department of Civil and Environmental Engineering, University of Washington, Seattle, Washington 98195, United States; ∥ Department of Earth and Planetary Sciences, University of California, Berkeley, California 94720, United States; ⊥ School of Public Health, 1438 University of California, Berkeley, California 94720, United States; # Department of Environmental Science, Policy, and Management, University of California, Berkeley, California 94720, United States; ∇ Department of Atmospheric Sciences, University of Washington, Seattle, Washington 98195, United States

**Keywords:** air pollution, environmental policy, inverse modeling, particulate matter

## Abstract

Air-quality standards are the backbone of air pollution regulation, yet they create an underdetermined planning problem: many emissions pathways can satisfy the same concentration target. The traditional approach to emissions mitigation design (“forward” scenario testing) evaluates candidate emissions-control strategies but does not directly determine the smallest set of emissions reductions sufficient to bring modeled concentrations below a standard. Here, we invert the planning task and solve a constrained Bayesian inverse problem to estimate the minimum, spatially explicit emissions changes required to meet a fine particulate matter (PM_2.5_) concentration target, while determining the distance to compliance across space, precursors, and sectors. We demonstrate the method with a case study of meeting the recently adopted annual primary National Ambient Air Quality Standard across the contiguous United States. Aggregate emissions reductions alone prove insufficient to predict attainment. Modeled strategies with similar total reductions range from perfect compliance to minimal progress, depending on which source locations, sectors, and precursors are controlled. Conversely, other plausible strategies increase the standard-compliant population fraction modestly but require substantially larger emission reductions. Our inverse method provides a benchmark for designing and evaluating air-quality attainment, while clarifying how spatial and sectoral levers drive progress toward a given concentration standard.

## Graphical abstract

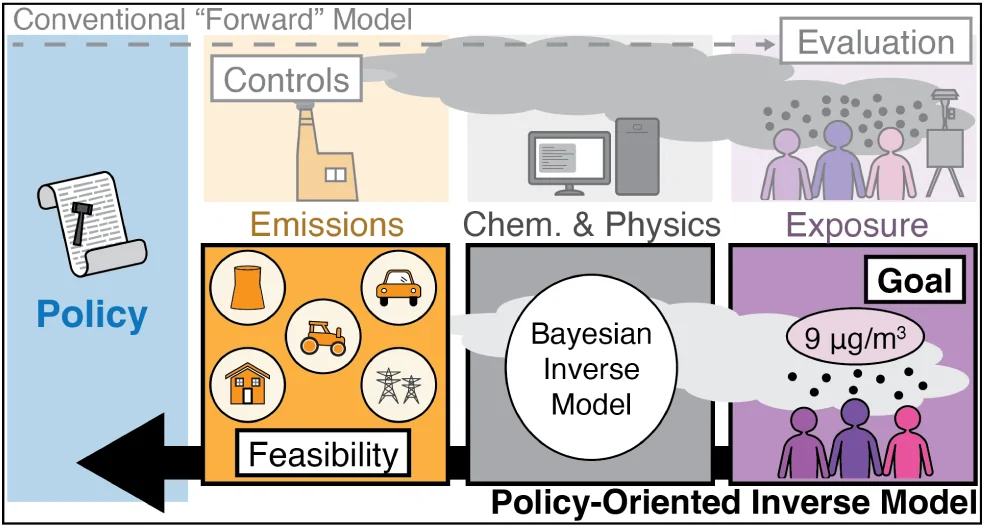

## 1 Introduction

Air pollution remains a leading environmental health risk worldwide. Regulatory systems frequently include health-based ambient concentration standards,^1,2^ for example, the National Ambient Air Quality Standards (NAAQS) established under the 1970 United States (US) Clean Air Act.^3–7^ In the US, states are legally required to design and implement emission-reduction plans to return air quality to compliance when exceedances are detected at regulatory monitors. A key challenge is that there are nearly infinite possible combinations of locations, sources, and pollutants that *could* be controlled. However, only a limited set of potential emissions controls can be reasonably translated into changes in ambient concentrations using “forward” atmospheric simulations. These scenarios are developed under incomplete information about emissions, source-receptor relationships, and future atmospheric conditions, as well as practical constraints related to available control technologies, regulatory authority, cost, and political feasibility.

We demonstrate an alternative approach to regulatory planning: What if one instead worked backward from air pollution standards to identify the minimum emissions intervention sufficient for attainment? Specifically, we adapt Bayesian inverse modeling, which is used in atmospheric science to reconcile estimated emissions with observational constraints,^8–13^ to the problem of identifying the minimum emissions intervention required to meet a concentration target. We define the “minimum intervention” as the smallest modeled reduction in controllable emissions that satisfies the specified concentration target given the constraints of atmospheric modeling. This benchmark is neither the least-cost nor easiest regulatory pathway; rather, it defines the idealized attainment requirement imposed by the modeled atmosphere, against which constraints of cost, technology, and authority can be compared.

To illustrate the potential of an inverse attainment-planning approach, we consider the problem of efficiently meeting the US annual “primary” (i.e., health-protective) NAAQS for fine particulate matter (PM_2.5_). We find that even modest emissions reductions of PM_2.5_ and its precursors (∼12%), when targeted toward the source locations, sectors, and precursors that most strongly influence nonattaining receptors, could produce the concentration changes needed for modeled attainment of the annual PM_2.5_ standard. Furthermore, this inverse policy modeling approach defines an air quality solution-space that could aid policymakers and researchers in defining and benchmarking candidate emissions controls.

Estimating the concentration changes associated with any individual scenario requires the ability to represent both the emissions changes and the relevant atmospheric processes. The compounding uncertainty created by estimating exposure responses to changes in emission inventories via chemical-transport modeling is a major limitation that this work does not attempt to address. Instead, we provide an alternative modeling framework that acknowledges these uncertainties and builds them directly into its design. Comprehensive chemical transport models (“CTMs”, e.g., CMAQ and WRF-Chem) predict spatiotemporal concentrations with the most detailed, process-based representations of atmospheric processes (e.g., advection, mixing, chemical transformation).^14,15^ However, these models and their inputs have their own uncertainties/biases, and their computational intensity limits the ability to rerun models across the large number of potential control strategies.^16–19^ Adjoint CTMs reduce this computational burden by efficiently calculating the sensitivity of a specified receptor, region, or scalar objective to emissions from all sources. A multireceptor attainment problem could be formulated as an adjoint-based objective, but the adjoint provides gradients of that objective rather than an explicit all-source to all-receptor transfer matrix and therefore generally requires iterative forward-adjoint optimization. Reduced-complexity models (e.g., InMAP, EASIUR, AP3)^17,18,20–23^ have enabled rapid exploration of many scenarios to support policy-relevant analysis,^17,23–34^ but it is still computationally laborious to determine which combinations of interventions yield the most mathematically efficient pathway to meet a concentration standard. Our approach instead uses a linearized all-source to all-receptor source-receptor operator to evaluate how emissions changes affect all receptors simultaneously and solve directly for the minimum intervention emissions reduction.

Given the numerous, multidimensional, and heterogeneous potential emission reduction pathways, NAAQS attainment is well-suited to optimization-based analysis. Many optimization methods are effective at solving multivariable problems.^35,36^ Early work in the 1970*s*/1980s compared distance to a target concentration against annual cost of attainment.^36–38^ Southern California was a testbed of this early work, with work minimizing ozone and particulate matter pollution in Los Angeles.^39,40^ Outside of academia, EPA regulatory impact analyses, state implementation planning, and related optimization studies have evaluated candidate control measures using costs, benefits, cost per unit concentration reduction, and other decision metrics.^41,42^ Multipollutant planning efforts have also solved for multiple objectives, including compliance, population exposure, air toxics risk, visibility, equity, and implementation feasibility.^43^ Recent work expanded upon these early optimization models, through least-cost multipollutant planning and benefit-cost air quality tools (e.g., OPERA, CABOT-O3).^44,45^ Yet these studies largely optimize over predefined control options or scenario ensembles, rather than solving directly for spatially explicit emissions adjustments across the full source-receptor system.

Rather than testing whether candidate controls meet the NAAQS, we solve backward from the health-based PM_2.5_ target (9 μg/m^3^), to identify the emissions-reductions required for compliance. For pollutants like PM_2.5_ that arise via primary and secondary formation, the spatial variation in source-receptor relationships is compounded by the diverse mixtures of precursor pollutants and emitting sectors. Rather than operating along a singular intervention axis (sector vs location),^24,29,46^ the model optimizes jointly on sector, pollutant, and location.^26–31^

This shift from forward scenario testing to inverse planning changes what atmospheric models can provide for regulatory design. The model no longer asks only whether a candidate control scenario is sufficient; it also identifies the minimum modeled intervention sufficient for attainment under the represented source-receptor relationships and control constraints. The resulting solution is best interpreted not as the *only* possible path to compliance, but as a *benchmark* for evaluating the efficiency and consequences of policy choices.

## 2 Materials and Methods

Our workflow mirrors conventional attainment planning but sets the regulatory goal as an explicit input. We then compare this inverse-derived minimum intervention against a large ensemble of forward scenarios and heuristic solutions to quantify how strongly attainment depends on the allocation, rather than only the magnitude, of emissions reductions.

Our approach is summarized in Figure 1. We begin with a baseline emissions inventory, *x*_*a*_, resolved by grid cell, precursor pollutant, release height, and economic sector across the contiguous US (see Section 2.1.2). Inverse modeling requires a linear operator; in order to adapt NAAQS compliance into an inverse modeling problem, we need to linearize atmospheric processes. The InMAP source-receptor matrix (ISRM), **H**, is used across the literature to linearly translate emission vectors into corresponding PM_2.5_ concentration fields, *y* = **H** *x*. We start from a baseline concentration field, *y*_*a*_, then specify a desired concentration end point, *y*_*o*_ (here: annual-average PM_2.5_ at or below 9 μg/m^3^) to solve for a controlled emissions field, *x*_*c*_, that satisfies this end point while minimizing reductions to baseline emissions under atmospheric constraints. The calculated emissions reductions are spatially explicit and resolved across five precursor species and six economic sectors. The result of our framework is a multidimensional minimal emissions intervention for attainment.

**Figure 1:**
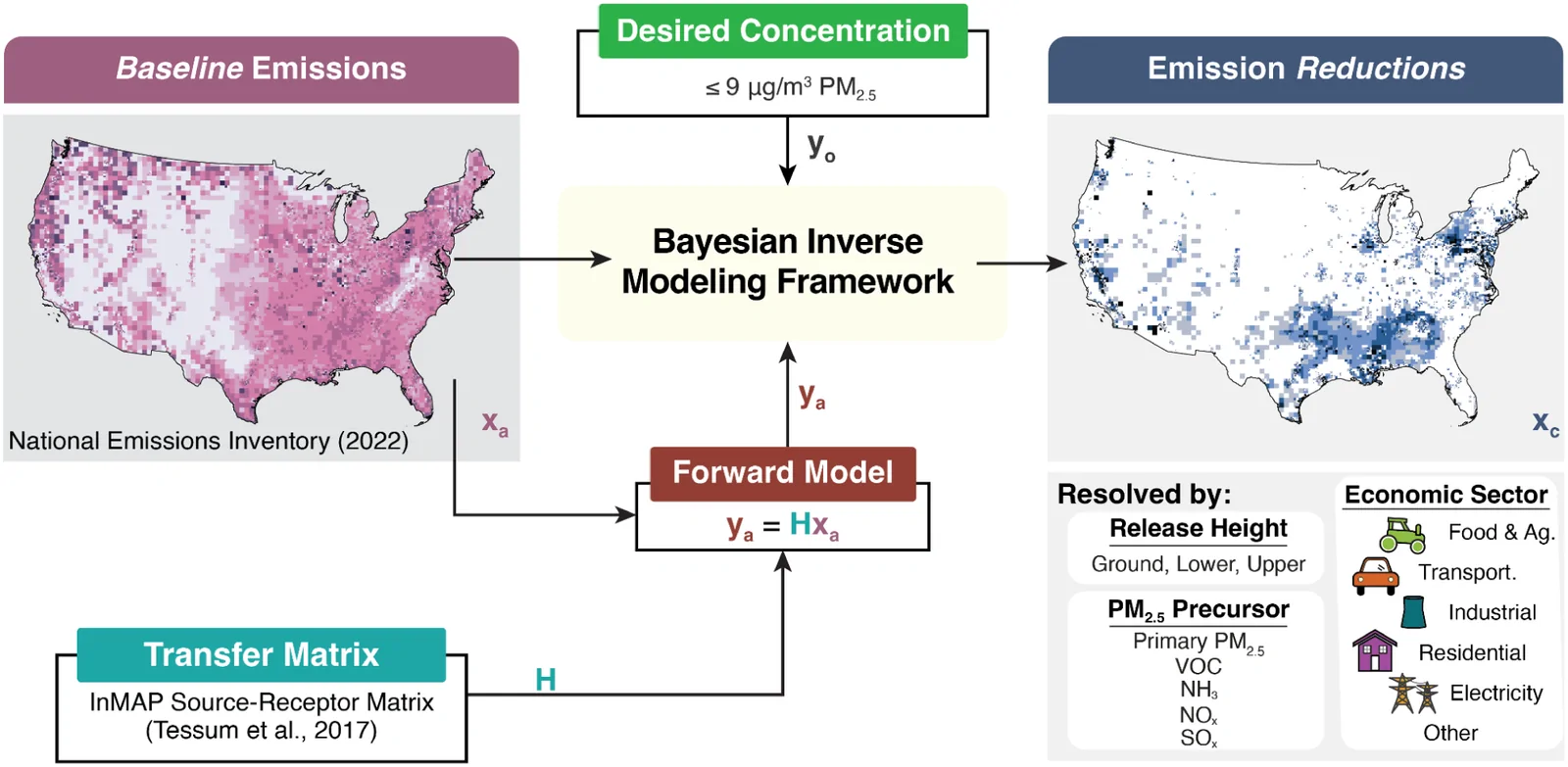
Model framework. We start from the baseline anthropogenic emissions field, x_a_, and use the InMAP source-receptor matrix H to compute the corresponding baseline concentration field, y_a_. The desired concentration end point, y_o_, (here: annual-average PM_2.5_ concentrations at/below 9 μg/m^3^) is imposed. Our model solves for a controlled emissions field, x_c_, that satisfies the target while minimizing deviations from baseline emissions under physical and health-protective constraints. Results are resolved across space (*n* = 54,111), five PM_2.5_ precursor species (primary PM_2.5_, NO_x_, NH_3_, VOC, and SO_x_) and six economic sectors.

### 2.1 Establishing Baseline Concentrations

#### 2.1.1 Atmospheric Source-Receptor Matrix

We estimate annual-average PM_2.5_ concentrations across the contiguous US using the ISRM, an existing source-receptor matrix that was built by running unit emissions in InMAP across a fixed irregular grid ∼157,000 times as part of Goodkind et al.^17,46,47^ The ISRM is not a perfect representation of the atmosphere, but it provides two fundamental features necessary for inverse modeling: it is linearized and captures complete, domain-wide source-receptor relationships.^17,46^ While ambient PM_2.5_ is the product of nonlinear atmospheric processes (e.g., chemistry, transport), the ISRM provides a first-order linear approximation of these processes.

We represent the linearized forward model:

1 y=Hx

where $y\in R^{n_{r}}$ is the vector of modeled baseline PM_2.5_ concentrations at receptors, $x\in R^{n_{e}}$ is the emissions vector, and $H\in R^{n_{r}\times n_{e}}$ is the ISRM. We solve for *n_r_* = 52,411 receptor cells (population-weighted grid from 1 to 2,304 km^2^). The emissions vector is resolved by horizontal grid cell (*n_e_* = 52,411), release layer (*n*_*cell*_ = 3), precursor pollutant (*n*_*pol*_ = 5), and economic sector (*n*_*sect*_ = 6), such that

$$n_{e}=n_{cell}\times n_{lay}\times n_{pol}\times n_{sect}=52,411\times 3\times 5\times 6$$

#### 2.1.2 Emissions Inventory and Correction

Emissions are derived from the 2022 National Emissions Inventory (NEI) and spatially allocated to the ISRM grid using SMOKE-analogous methods.^48^ The five PM_2.5_ precursor species include primary PM_2.5_, oxides of nitrogen (NO_*x*_), ammonia (NH_3_), volatile organic compounds (VOC), and oxides of sulfur (SO_*x*_). The six economic sectors are Electricity, Food and Agriculture, Industrial, Residential, Transportation, and Other (including controlled burning). Emissions that contribute to baseline concentrations but are not anthropogenic (wildfire-related, biogenic, soil NO_*x*_) are included as a seventh “uncontrollable” category that is in the baseline but held fixed during optimization. In the core analysis, emissions from all anthropogenic sectors are eligible for 100% reduction. This is an upper-bound benchmark and does not imply that all anthropogenic emissions can be reduced using existing control strategies. More generally, sector-, precursor-, or source-specific controllability can be represented by excluding emissions from the control vector, imposing maximum reduction fractions, or assigning larger prior penalties. We demonstrate this flexibility in the agricultural-control exclusion sensitivity, described in Section 3.3. Thus, the inverse solution identifies only the reductions within the specified control vector required to achieve attainment in the presence of an irreducible background burden (Figure S1, SI Section S5).

To ensure that the inverse model is targeting real-world concentration exceedances, we applied an additional baseline-correction step that uses an analogous workflow (see Figure 1) and satellite-derived, model-assisted 2022 PM_2.5_ fields^49^ to estimate observation-informed baseline concentrations and emissions. Because the attainment inversion is solved based on where exceedances exist at the baseline, the resulting reduction pattern is conditional on the baseline emissions inventory. Uncertainty in emissions magnitude, spatial allocation, or sector assignment affects where and by how much the inverse model will preferentially reduce emissions. The satellite-informed correction step reduces bias in the baseline concentration state, but it does not eliminate emissions inventory uncertainty. The inverse solution presented here is conditional on the corrected baseline. This satellite-derived data set also allows us to test the robustness of conclusions to bias in the baseline state. The resulting corrected baseline and its differences from the uncorrected inventory are shown in Figures S2–S5. Further details on the emissions processing, sector mapping, satellite-informed baseline correction, and associated uncertainty treatment are provided in SI Section S1 and Figures S1–S6.

### 2.2 Bayesian Inverse Formulation for Attainment Planning

Traditional Bayesian inverse modeling reconciles observed and modeled concentrations by estimating emissions adjustments that best fit measurements while remaining close to a prior emissions estimate and avoiding large changes in areas with high observational uncertainty. Here, we outline how we adapt this modeling framework for policy planning. The full formulation is provided in SI Sections S1–S2.

#### 2.2.1 Target Construction as a Flexible Attainment Constraint

A rigid target that forces every receptor to *exactly* 9 μg/m^3^ annual-average concentration is physically unrealistic with compensatory emissions increases prohibited. Instead, we implement a flexible, iterative constraint for attainment: at each iteration, exceeding receptors in the current modeled field are capped to the standard, while receptors already at or below the standard are not penalized for remaining below nor improving further. We make an exception for two grid cells that exceed the standard due to uncontrollable emissions alone (Figure S1). This target enforces a dynamic upper bound without imposing a rigid floor (Figure S7; SI Section S2.2). A hard equality-style target would penalize further reductions at receptors already below 9 μg/m^3^, even when those reductions are needed to bring nearby receptors into compliance (Figure S8).

#### 2.2.2 Log-Space Emissions Updates and Constrained Inverse Formulation

We solve for emissions changes relative to a baseline estimate *x_a_*. To ensure that emissions cannot increase and to represent controls as multiplicative reductions, we work in log space:

$$\theta =\ln\left(\frac{x}{x_{a}}\right);,x=x_{a}⊙\exp\left(\theta \right)$$

where ⊙ denotes elementwise multiplication. We enforce no emission increases by restricting θ ≤ 0. We estimate θ via maximum a posteriori inference by minimizing:

2 $$J\left(\theta \right)=\frac{1}{2}{\left(y_{o}-H\left(x_{a}⊙exp\left(\theta \right)\right)\right)}^{T}S_{o}^{-1}\left(y_{o}-H\left(x_{a}⊙\exp\left(\theta \right)\right)\right)+\frac{1}{2}\theta^{T}S_{\theta }^{-1}\theta$$

Here, **H** represents the linearized relationships between emission changes at all sources and concentration changes at all receptors (Section 2.1.1), **S**_o_ is a receptor-space covariance encoding tolerance or uncertainty in concentration-space deviations from the target, and **S**_**θ**_ is an emissions-space covariance encoding the relative cost of deviating from baseline emissions. In practice, **S**_o_ prevents the inversion from prescribing large reductions that “force” compliance in receptors where the modeled source-receptor response is comparatively uncertain. By contrast, **S**_**θ**_ governs how readily emissions in different locations, precursors, and sectors can be adjusted. As described in Section 2.1.2, controllability and implementation constraints can be represented through control masks, reduction limits, or penalty weights. Here, we allow emissions to decrease up to 100% of baseline, with **S**_**θ**_ governing the relative penalties that determine where and how reductions are allocated. We assume **S_o_** is diagonal, meaning that tolerance for target mismatch is independent across receptors. In contrast, **S**_θ_ includes off-diagonal elements to link emissions adjustments together, as an activity-based control assumption: if activity in a given sector is reduced at a given location, then all precursor emissions from that sector-location combination tend to decline together rather than being adjusted independently.

### 2.3 Benchmarking Attainment Efficiency

Many distinct combinations of precursor-sector-spatial controls can achieve the same concentration target. To quantify why allocation matters and to contextualize the inverse-derived minimum-intervention benchmark, we benchmark against a large ensemble of forward scenarios.

#### 2.3.1 Forward Scenario Ensemble

We perform a Monte Carlo experiment that generates one million emissions reduction scenarios and evaluates their concentration outcomes using the baseline emissions x_*a*_ and same source–receptor operator **H**. Each scenario applies randomized independent reductions (in 1% increments) across 52,411 grid cells and six emissions sectors, with Latin Hypercube Sampling to efficiently span a high-dimensional control space. This simplified ensemble reveals the dispersion of attainment outcomes among strategies with similar aggregate emissions reductions. These scenarios are not samples from the inverse posterior distribution; rather, they form a forward exploratory ensemble bounding a broader space of alternative control pathways. Additional details on the forward solution-space ensemble and ensemble size sensitivity are provided in SI Section S3.1 and Figure S9.

#### 2.3.2 Attainment Outcomes and Mitigation Burden

For each emission-reduction scenario, we quantify compliance benefits and mitigation burdens. We refer to the reduction in the population remaining above the annual PM_2.5_ standard as the compliance benefit of a scenario. This metric measures progress toward the regulatory threshold used in this study but does not imply that PM_2.5_ health effects vanish below 9 μg/m^3^ or that further reductions lack value. Reducing any PM_2.5_ exposure is beneficial; therefore, we also quantify population exposure distributions as a compliance benefit. Notably, our compliance benefits differ from the true implementation design of the NAAQS which is enforced based on measured concentrations at discrete monitors, whereas our inverse solution reduces modeled concentrations across the continuous receptor grid. We quantify the mitigation burden as the compliance penalty associated with misallocated controls by comparing the additional emissions reductions required to achieve the same attainment outcome relative to the one million forward scenario ensemble.

Because emissions are resolved by precursor species, aggregate emissions changes are not comparable across sectors or candidate solutions in native mass units alone. We express emissions changes in primary PM_2.5_-equivalent units. This metric combines primary PM_2.5_ and precursor emissions by casting them into equivalent units to compare solution pathways. We construct these units for each precursor by calculating the population-weighted total PM_2.5_ exposure produced by an individual grid cell and precursor and estimating the amount of primary PM_2.5_ that would need to be emitted to produce that identical signal. This formulation is analogous to the concept of the carbon dioxide equivalent unit, which is alternatively used to normalize the global warming potential of different greenhouse gases. These PM_2.5_-equivalent quantities are used only as summary metrics for interpretation and comparison; all forward concentration calculations and inverse optimization are performed using the full precursor-resolved emissions representation. Further details on the construction of the primary PM_2.5_-equivalent metric are provided in SI Section S4.

## 3 Results and Discussion

Our results highlight how highly structured, heterogeneous, and multidimensional the attainment problem is. First, we illustrate the spatial relationships between the baseline concentration exceedances and emissions. Second, we juxtapose our solution atop the forward ensemble to show that similar total reductions can yield sharply different compliance benefits, and that poor allocation of emission-reductions carries a substantial mitigation penalty. Third, we present the spatially explicit and sectorally resolved benchmark emission reduction proposed by inverse model solution, assuming linear atmospheric approximations, and discuss the differential burden of compliance nationwide.

### 3.1 A Heterogeneous Attainment Landscape

We begin with the baseline conditions (Figure 2). The observationally corrected baseline total PM_2.5_ concentrations (Figure 2a) show that exceedances are neither spatially uniform nor proportional to the emissions inventory. In the 2022 baseline conditions, the majority of the contiguous US is below the annual PM_2.5_ standard; exceedances are concentrated in a limited set of high-exposure regions (e.g., southern California, California’s Central Valley, the Gulf Coast, other scattered urban/industrial corridors). Thus, the national attainment problem is driven by a relatively small subset of receptors.

**Figure 2:**
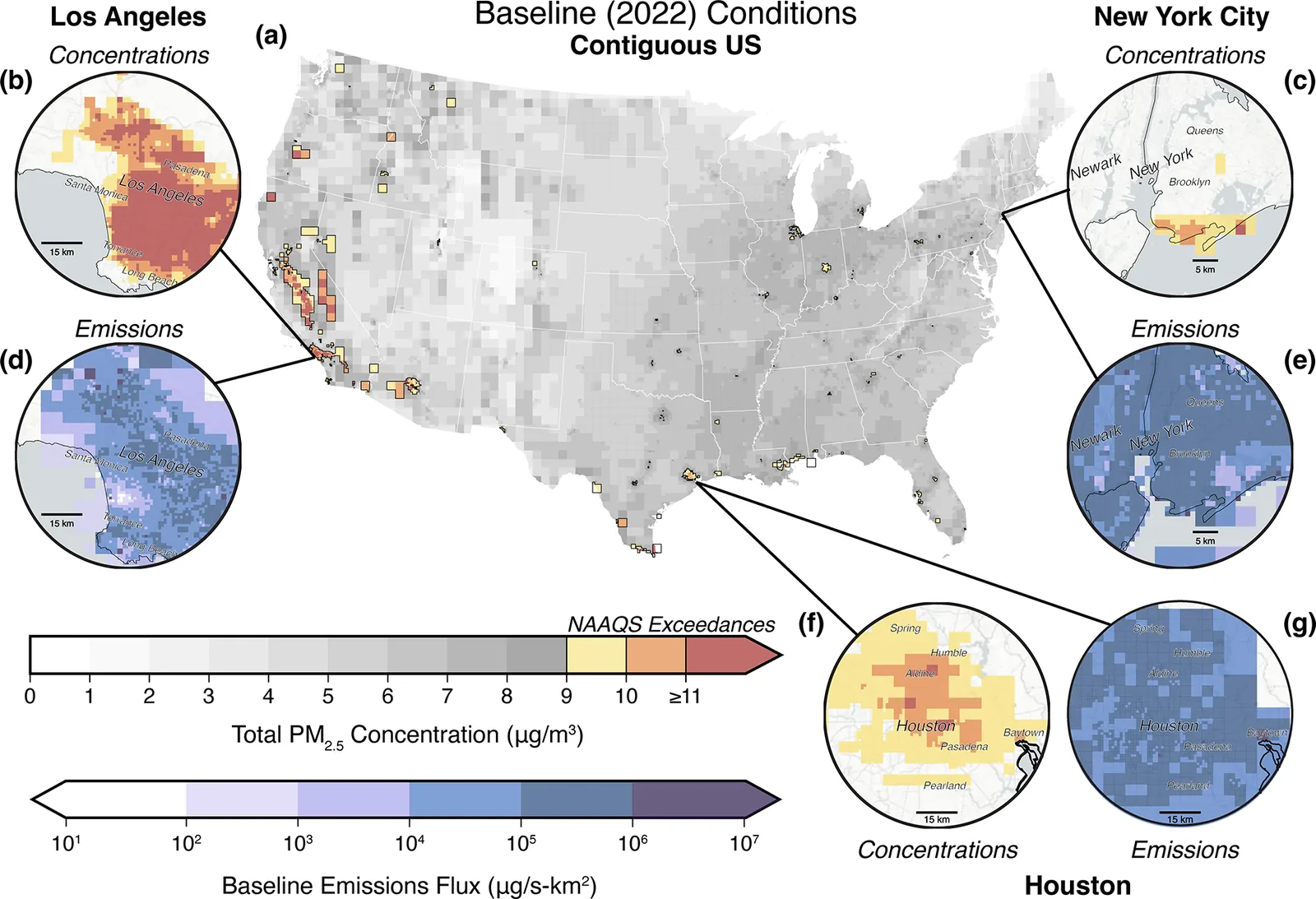
Baseline 2022 PM_2.5_ concentrations and emissions across the contiguous US demonstrate national heterogeneity in air pollution. Results are shown on a variable-resolution grid, with finer grid cells in more densely populated areas. (a) Gray tones denote concentrations below the annual PM_2.5_ NAAQS; outlined warm colors denote exceedances above 9 μg/m^3^, with darker tones indicating progressively higher concentrations. (b, c, f) Urban concentration insets for Los Angeles, New York City, and Houston, respectively. To highlight areas relevant to attainment, these insets show only grid cells exceeding 9 μg/m^3^. (d, e, g) Corresponding baseline (primary PM_2.5_-equivalent) emissions fluxes aggregated across modeled anthropogenic sources, shown on a logarithmic scale for the same three cities.

The spatial distribution in exceedances matters because attainment is governed by the upper tail of the receptor distribution rather than by national-average concentrations. For example, a strategy that reduces emissions broadly without targeting the high-leverage receptors may substantially reduce emissions with little compliance benefit. Conversely, a more targeted strategy could approach attainment with a smaller total intervention. In this sense, Figure 2 suggests why forward scenario testing alone can be inefficient. When noncompliance is generated by a subset of receptors, the value of a unit emissions reduction depends strongly on where in the system it occurs and which precursor is reduced.

The urban insets illustrate this idea by demonstrating how spatial patterns in emissions and concentrations can vary. Los Angeles, New York City, and Houston are all regions of intense baseline emissions, yet their modeled concentrations patterns differ substantially (Figure 2b, c, and f). In Los Angeles and Houston, exceedances are broad and spatially contiguous across the metropolitan basin (Figure 2b). In New York City, exceedances are narrower and more spatially confined, despite similarly intense emissions. While these concentration insets aim to illustrate spatial patterns rather than precisely quantify concentrations, InMAP has previously been applied to city-scale analyses of annual PM_2.5_ concentrations and emission interventions,^50–52^ and the baseline concentrations used here are corrected using the satellite-derived PM_2.5_ fields described in Section 2.1.2. We have not conducted a separate city-specific evaluation of these concentration fields against independent high-resolution data sets. Accordingly, the urban panels are used to illustrate annual-average patterns relevant to the attainment analysis rather than detailed neighborhood-scale air-quality patterns; higher-resolution and higher-fidelity studies provide complementary characterization of local concentration variability.^53–56^ The patterns shown here reflect the combined source-receptor relationships represented in the ISRM, including transport, dispersion, and chemistry. Because these processes are embedded collectively within the reduced-complexity operator, we do not attribute the differences across cities to any single atmospheric mechanism. Instead, these contrasts highlight how emission-attainment relationships are mediated by topography, atmospheric transport, local dispersion, and the secondary formation chemistry. Thus, high emissions do not necessarily produce extensive noncompliance, and the potency of an intervention cannot be inferred from source intensity alone.

The emissions panels reinforce this mismatch. Baseline emissions flux spans roughly 6 orders of magnitude across the domain (Figure 2d, e, and g; see also Figures S2–S3 for national baseline emissions fields and Figure S6 for the state-level baseline decomposition), with strong clustering in metropolitan areas, transportation corridors, industrial regions, and agricultural zones. This suggests a central asymmetry for pollution attainment: air-quality standards are defined in concentration space, but interventions operate in emissions space, and the mapping between the two is highly nonuniform. The baseline reveals that attainment planning is fundamentally a problem of heterogeneous influence where some sources matter more than others for the receptors that determine compliance.

The baseline also suggests that the attainment problem is intrinsically multiscalar. Some exceedances are associated with dense urban source mixtures, whereas others reflect broader regional contributions accumulated through transport and secondary formation. Attainment is likely not reducible to a purely local problem or to a uniform national reduction. It is a coupled problem in which the relevant control levers depend jointly on location, precursor class, and source type.

### 3.2 Inverse Model Identifies Efficient Frontier of Compliance

Even under our simplified setup, the forward design space is too large to enumerate exhaustively: with 52,411 grid cells and 6 anthropogenic sectors, there are 314,466 distinct location-sector combinations. Allowing each control to vary from 0–100% in 1% increments creates a combinatorially vast space, such that the 10^6^ scenarios shown here represent a necessarily small but representative sampling of feasible allocations (Figure S9). This ensemble exemplifies how attainment depends more strongly on how/where emissions are reduced rather than aggregate magnitude.

In Figure 3, we juxtapose various algorithmic solutions atop the forward solution space. The baseline conditions (Figure 2) are the bottom-left white point on Figure 3a. Strategies with similar anthropogenic emissions reductions yield a wide range of compliance benefits (Figure 3a). Importantly, there is no one-to-one relationship between total reduction and compliance: the relationship is nonlinear. The nonlinearity arises because the specific location-sector reduction combinations have distinct marginal benefits toward attainment. Because the *x*-axis includes only anthropogenic, controllable emissions (∼70% of total baseline emissions), we compare scenarios against differences in the deployment of the controllable share of the system (see Figure S1).

**Figure 3:**
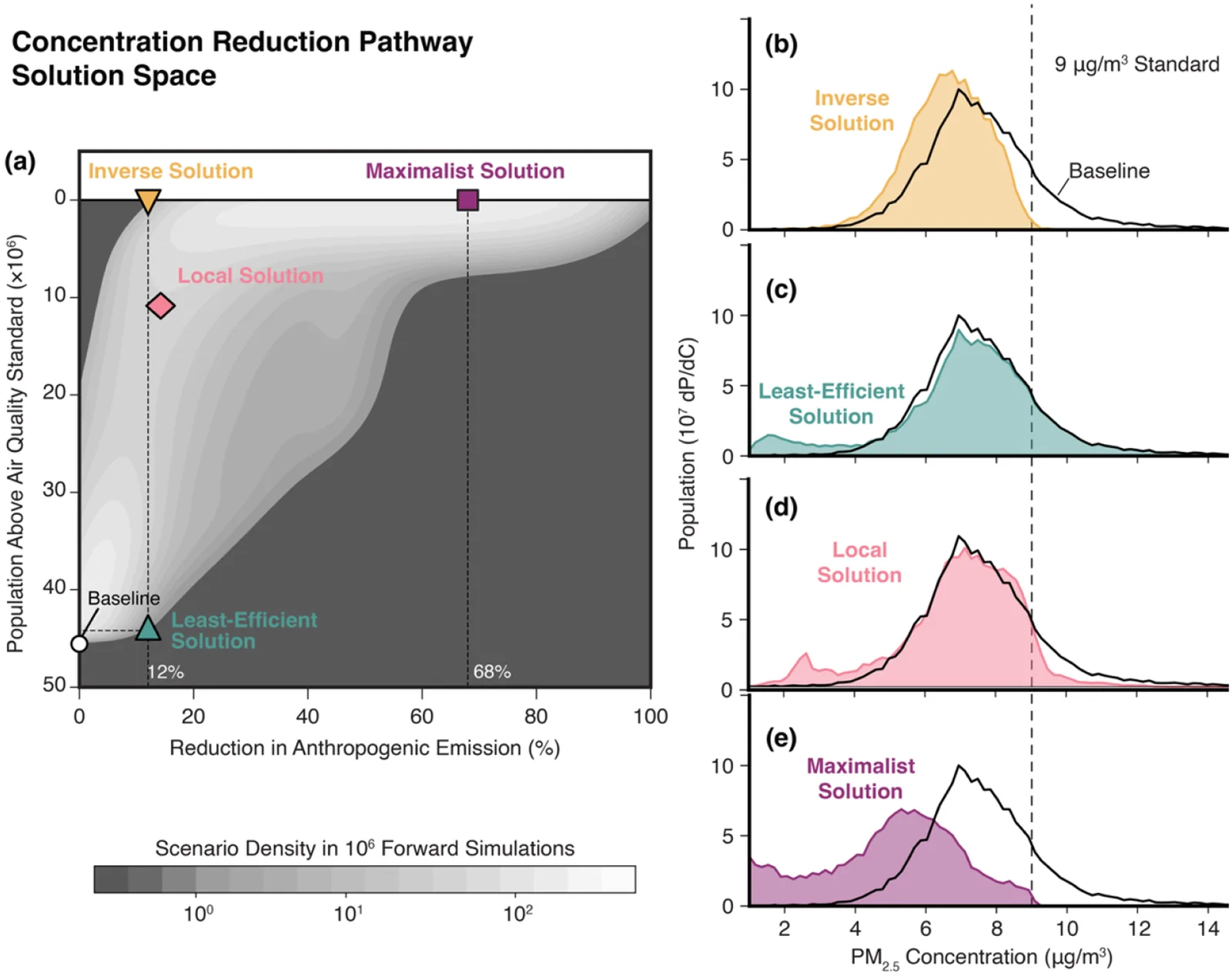
NAAQS compliance occupies a narrow efficient frontier in emissions-control space. (a) Density of 10^6^ forward simulations in the space of total anthropogenic emissions reduction and population remaining above the annual health-based PM_2.5_ standard. Each simulated point represents a distinct spatial-sectoral-magnitude allocation of emissions reductions. Contours summarize the density of scenarios occupying this space. The *x*-axis reflects reductions in controllable emissions only. Baseline conditions are represented as a white circle with black outline. The inverse solution (gold triangle) lies on the efficient frontier, achieving compliance at ∼12% aggregate emissions reduction. Three additional scenarios represent poor spatial and sectoral allocation (teal triangle: “Least Efficient Solution”), a simple local reduction scheme (pink diamond: “Local Solution”), and brute-force heuristically chosen reductions (purple square: “Maximalist Solution”). (b–d) Population-weighted distributions of PM_2.5_ exposures for each scenario are compared to the baseline distribution and the annual NAAQS (dashed vertical line).

The upper bound of the gray-shaded solution space (Figure 3a) defines the most efficient frontier: the set of strategies that delivers the greatest compliance benefit possible for a given aggregate emissions reduction. The inverse solution sits on the elbow of this frontier: it achieves compliance at ∼12% total anthropogenic emissions reduction, suggesting that the standard could be met with a relatively modest reduction in controllable emissions when optimally allocated.

By contrast, a scenario with a poorer allocation of nearly the same total anthropogenic reduction (“Least Efficient Solution”, the upward teal triangle) leaves ∼45 million people above the standard. This comparison highlights that attainment is governed by *which locations* and *which sectors* reduce emissions, not simply *by how much* is reduced in aggregate. The difference in compliance outcomes arises because their reductions are placed in different parts of the source-receptor system (Figures S10–S11). This does not imply that such reductions have no value. A scenario can be inefficient for achieving modeled attainment while still beneficially reducing exposure for an individual population or otherwise advancing another environmental goal (e.g., visibility). This scenario is inefficient with respect only to the attainment objective.

We contrast these scenarios against two simpler, more interpretable algorithms. The “Local Solution” (pink diamond in Figure 3a, spatial pattern in Figure S12a) eliminates all controllable emissions within grid cells that exceed the NAAQS. This strategy removes ∼14% of controllable emissions, yet leaves ∼11.9 million people above the NAAQS. It requires ∼2% more emissions reduction than the inverse solution while protecting ∼11.9 million fewer people from exceedance. The “Maximalist Solution” (purple square in Figure 3a, see SI section S3.2 for algorithmic details and Figure S12b for the resulting spatial reduction pattern) is a sequential heuristic referred to as “greedy approach” by the optimization literature that, at each step, selects the emissions reduction that produces the largest immediate improvement in attainment.^57^ We refer to this as “Maximalist” because each step chooses the best available reduction based on the current state without optimizing the full sequence of reductions jointly.^35,57^ This scenario is analogous to the optimization algorithm employed in Wang et al.^29^ and similar to that used by the US EPA in the 2006 NAAQS Regulatory Impact Analysis.^42^ In Figure 3a, the “Maximalist Solution” achieves compliance only after ∼68% emissions reduction, which is a five- to six-fold larger reduction in controllable emissions for the same compliance outcome. Because it proceeds sequentially rather than solving the problem globally, it runs into a thresholding problem. Early steps capture the most obvious marginal gains, but those gains diminish as the remaining noncompliance becomes concentrated in a harder-to-reach subset of receptors. The algorithm requires progressively broader and less efficient reductions to eliminate the remaining exceedance. In comparison, the inverse solution avoids this problem by optimizing across the full system simultaneously, balancing additional emissions reductions against progress toward simultaneous compliance across grid cells.

Figure 3b–e depict the differences across the population exposure distributions. The baseline distribution (black lines) contains a substantial right tail above the 9 μg/m^3^ threshold. The inverse solution completely removes the upper tail of the exposure distribution to move the population experiencing exceedances below the standard (Figure 3b). The “Least Efficient Solution” (Figure 3c) shifts the lower/cleaner tail of the distribution only, despite a similar emissions reduction. The “Local Solution” (Figure 3d) eliminates the most extreme tail of the population distribution but fails to achieve the goal. The “Maximalist Solution” also brings the upper end of the distribution below the threshold, but only after a much larger overall leftward shift (Figure 3e), consistent with broad overcontrol of the system. While there is a case to be made that the “Maximalist Solution” provides larger-scale population health benefits, it does so with a substantially higher mitigation burden. If the policy goal were to maximize health benefits or total exposure reduction, this broader pathway could be more attractive than the minimum-intervention pathway. That is a different objective from the central attainment problem posed here.

These exposure distributions reveal that the inverse solution reflects a distinct logic from other optimization methods. Rather than reducing concentrations broadly across all receptors or dramatically in only a subset of receptors, it targets the portion of the receptor distribution that determines compliance. This distinction follows from the attainment objective posed here, but it should not be interpreted as a health-based threshold claim. PM_2.5_ reductions below the standard can still produce health and economic benefits, and broader national policies may generate knock-on benefits in already-compliant locations. Decision-makers therefore face a trade-off between mitigation burden and exposure reduction: broader controls may produce larger total exposure or health benefits but require more emissions reduction than the minimum-intervention pathway needed for modeled attainment. Our solution identifies the smallest modeled intervention that removes annual-standard exceedances while providing a benchmark against which broader exposure-reduction, benefit-maximizing, or equity-focused strategies can be compared. That distinction is especially important for annual-average PM_2.5_, where the policy objective is defined by threshold exceedance rather than by minimizing concentration everywhere. Under such a standard, reductions applied to already low-concentration or low-leverage portions of the system may have little effect on attainment, whereas smaller reductions applied to threshold-relevant receptors can be much more effective.

Overall, Figure 3 shows that the attainment problem contains a large set of emission reduction solutions but a much smaller *efficient* subset. Forward scenario analysis can sample the feasible set, but it does not naturally identify the efficient frontier. The inverse solution’s value is not that it identifies the *only* possible path to compliance, but that it establishes a reference point against which the compliance penalty of alternative strategies can be measured. The inverse solution can provide a starting point for forward modeling with higher fidelity modeling tools for feasibility and implementation. These recommendations are not necessarily practical along our grid but even applying the aggregated state-and-sector reduction recommendations to individual gridded emissions yields dramatic benefits to exposure (Figure S13).

### 3.3 Attainment Requires Diverse Reductions across States and Sectors

The inverse solution identifies the spatial and sectoral distributions of emission reductions required to achieve national compliance. Although baseline exceedances occur in a limited set of receptor locations, the corresponding inverse solution is broader. A receptor above the annual PM_2.5_ standard may be influenced by emissions from many upwind grid cells, precursors, and sectors, particularly where secondary formation and regional transport contribute to nonattainment. Thus, a spatially concentrated concentration problem can correspond to a more spatially distributed emissions-control problem and the inverse solution includes reductions outside of the grid cells exceeding the standard.

Meeting the health-based NAAQS for PM_2.5_ requires a total reduction of ∼33 kg/s of anthropogenic emissions across the contiguous US (Figure 4). These reductions should be interpreted as the source-space response for modeled attainment, not simply as controls located at the nonattainment receptors themselves. Agriculture dominates across broad areas of the West, Central, and Southeast US, while industrial, transportation, electricity, and other sources dominate elsewhere (Figure S14a). PM_2.5_ concentration reductions exhibit a different sectoral pattern (Figure S14b), illustrating that the sector with the largest emission reduction does not necessarily produce the largest local air-quality improvement. Figure 4 shows the cumulative inverse model solution in primary PM_2.5_-equivalent units; precursor-resolved state-by-state decompositions are shown in Figures S15–S19. At the national scale, this reduction is distributed across several sectors: agriculture ∼35%, industrial ∼23%, and transportation ∼18%. Thus, the minimum-intervention pathway to attainment must be cross-sectoral.

**Figure 4:**
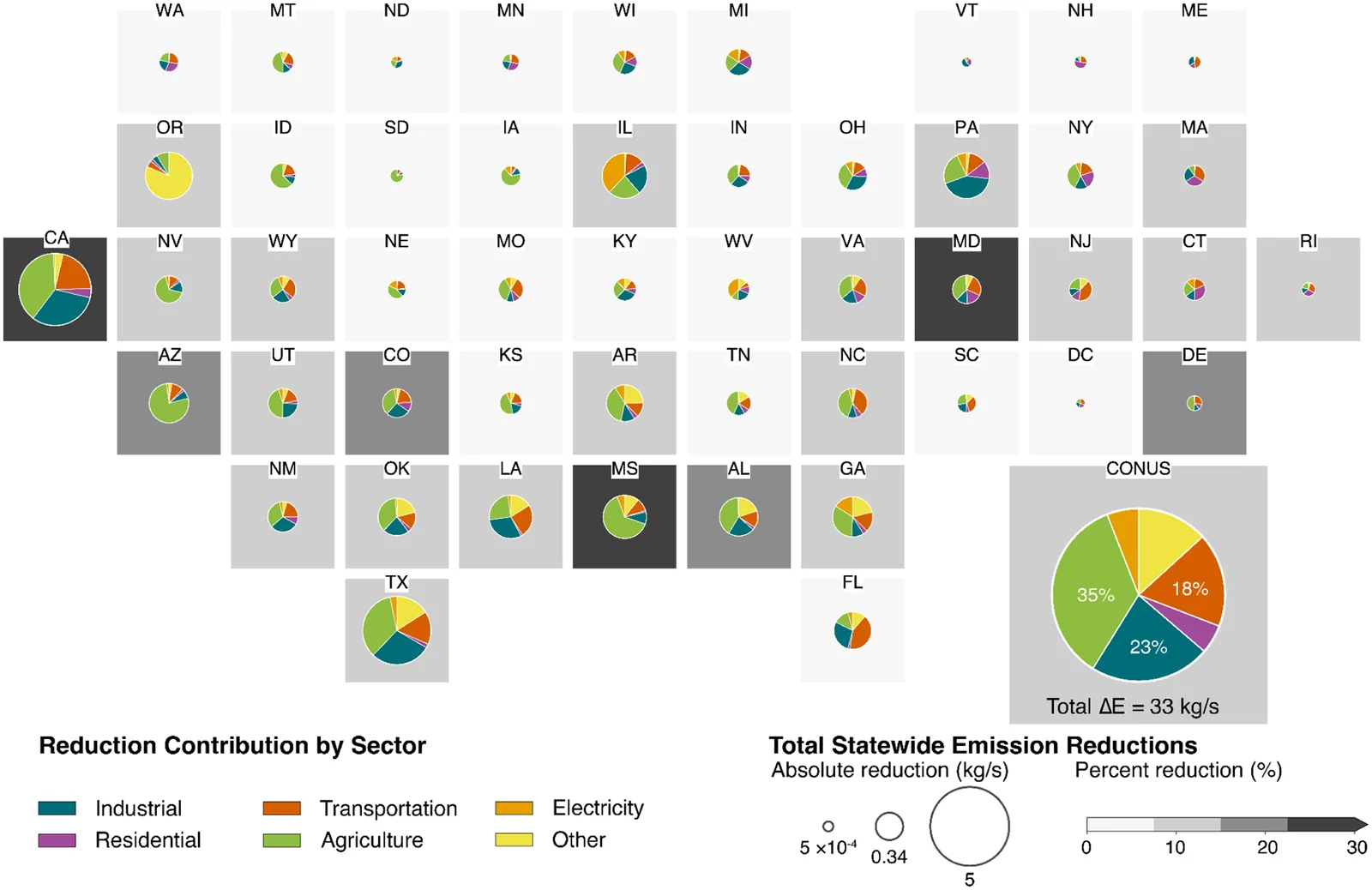
Sectoral emissions reductions required by the inverse model solution. Each tile represents state-level aggregate emission reductions. The pie-chart area scales with the absolute emissions reduction required in that state (kg/s); tile shading indicates the percent of emissions reduction relative to that state’s baseline emissions. Wedge colors denote the sectoral contribution to each state’s required reduction. A nationwide summary is shown at the bottom right. Equal-sized pies are shown in Figure S20.

National totals mask substantial geographic heterogeneity, as shown in the state-by-state tiles in Figure 4. Similarly, state totals mask finer grid-cell heterogeneity within states. Precursor-resolved state-level decompositions are shown in Figures S15–S19, and Figure S20 presents the sectoral decompositions using uniformly sized pie charts for easier visual comparison of dominant sectors. The largest absolute reductions are concentrated in a relatively small number of states like California and Texas. Additional large reductions are required in parts of the Gulf Coast, south-central US, and the eastern industrial corridor. This pattern is broadly consistent with Figure 1, but the state-level decomposition demonstrates that neither emissions magnitude alone nor the presence of exceedances determine a state’s importance in the attainment solution. A baseline reference for the state-level inventory is shown in Figure S6.

To understand the difference between the spatial pattern of baseline emissions and required reductions, it is useful to compare reductions in absolute (pie size) and relative (shading) emissions. Maryland and Mississippi, for example, show comparatively high relative control requirements despite much smaller absolute reductions than California or Texas. This indicates that a state can play a large role in the national attainment solution because it emits a lot, because its emissions strongly affect noncompliant receptors, or both.

The sectoral composition of the state-level reductions also varies substantially. Some states show broad multisector reductions, indicating that several source classes contribute materially to noncompliance. California and Texas, for example, require substantial agricultural, industrial, and transportation emission reductions. The significance of this result is not that these sectors are major baseline contributors to PM_2.5_, which is already well established. Rather, the inversion identifies the marginal role of each sector within the benchmark attainment pathway. Figure S14 reinforces the idea that the sector receiving the largest emission reduction in a location does not necessarily produce the largest local concentration reduction. Other states focus more on individual source sectors. For example, Pennsylvania requires predominantly industrial sector reductions, while parts of the southwest and south-central US require large agricultural emission reductions. Oregon has anomalously high reductions from the “Other” category, which is due to high baseline emissions from prescribed burning activities contributing to exceedances in-state and in California. These differences demonstrate how the control burden for a national concentration target depends on the overlapping spatial patterns of sources, regional transport, and population distributions.

We run a second case where agricultural emissions are held fixed to illustrate how restrictions on the admissible control space alter both the composition and feasibility of the attainment pathway (Figure S21). In this case, the inverse solution reallocates reductions toward industrial, transportation, and other sources. However, the remaining sectors cannot fully replace the lost agricultural leverage: the national emission reduction decreases from 33 to 25 kg/s, while the population remaining above the annual PM_2.5_ standard increases to ∼6.1 million. The smaller aggregate reduction does not indicate a less burdensome pathway; rather, it reflects locations where the minimum concentration attainable through controls on the remaining sectors is still above the standard. The core and no-agricultural-control cases provide bounding assumptions rather than precise technology-feasible scenarios; partial agricultural controllability would be expected to produce outcomes between these limits. This counterfactual provides information beyond conventional source attribution: agricultural emissions are not only substantial contributors to PM_2.5_ but also cannot be substituted for by making reductions from other sectors.

While the Clean Air Act includes provisions addressing interstate transport and downwind pollution, implementation still occurs through jurisdictional planning processes that translate regional source-receptor relationships into enforceable control strategies. Notably, to achieve national compliance, emissions reductions must occur in every state. Some states are an important part of the solution because they have noncompliant receptors, others because their emissions contribute to noncompliance in downwind states. This pattern reflects the central asymmetry for attainment: compliance is measured locally whereas the emission reductions needed for attainment are often regional for pollutants like PM_2.5_.

More broadly, the state-level decomposition suggests that attainment planning is not simply a matter of reducing emissions wherever emissions are largest. It is shaped by source-receptor relationships, regional transport, and the spatial distribution of exceedances. The inverse solution connects these pieces by translating a receptor-defined standard into a set of emissions reductions resolved simultaneously across geography and sector.

## 4 Implications and Future Work

Our illustrative application of inverse modeling to the NAAQS suggests a reimagined air quality planning process. The central challenge is not determining how much to reduce, but *which* emissions changes most efficiently achieve the concentration standard. The spatial heterogeneity of emissions and concentrations in the baseline (Figure 2) results in a wide solution space in which strategies with similar total emissions reductions can yield radically different outcomes in the context of meeting air quality standards (Figure 3). The inverse solution identifies the optimal structured set of reductions distributed across, space, sectors and precursor emissions (Figure 4) that would otherwise be difficult to find iteratively in a forward modeling framework. These results highlight the broader emissions-concentrations asymmetry: emissions reductions are implemented at the source, but air quality standards, health impacts, and disparity outcomes are evaluated at receptors. As a result, equal reductions in emissions mass can produce different concentration changes and therefore emission reductions cannot be used as proxies for receptor-based outcomes.

For a number of reasons, the inverse solution we presented in Figure 4 should not be interpreted as a prescription for implementation. The solutions derived here are mathematically optimal within the modeling framework and governing equations. As suggested in previous work, we recommend that the inverse model solution serves as a starting point that should be rigorously evaluated with higher fidelity tools.^41,43^ The inverse model’s guidance also may not address other relevant considerations such as regulatory authority, jurisdictional fragmentation, technological availability, political feasibility, and implementation cost. Future models can build in some of these feasibility-informed constraints. Still, by quantifying the sector-precursor-location mixture for the minimum-intervention pathway, the inversion provides an informed starting point for designing feasible compliance strategies.

Here, we demonstrate our proposal for a reimagined air quality planning process with an illustrative example application in California. Our inversion suggests that emissions reductions from agricultural and industrial sources contribute the largest share of the minimum intervention pathway in California (Figure 4). These sectors are important within the modeled attainment problem and within the boundaries of model-emission inventory uncertainty but may differ substantially in regulatory feasibility and control cost. Californian regulators could use this minimum intervention benchmark to prioritize scenario development in those source sectors and locations. They can then evaluate these candidate emission reduction scenarios with higher fidelity, process-resolved CTMs to compare against the inverse benchmark. As shown here, the inverse modeling approach should serve as the first stage of planning rather than the last.

### 4.1 Methodological Considerations

There are several limitations of this analysis that warrant consideration; these are described in detail in SI Section S6 and summarized here. The benchmark is conditional on both the transfer operator (**H**) and the baseline inventory, so errors in transport, chemistry, emissions magnitude, spatial allocation, or sector assignment can shift the detailed solution. The ISRM provides an explicit annual-average linear operator suitable for national screening and has been evaluated and applied extensively in previous air-quality and exposure analyses.^24,28,29,46,47,58^ However, it does not resolve seasonal episodes, nonlinear chemistry, or local gradients with the fidelity needed for final regulatory decisions. We represent uncertainty in the target and emissions prior through **S**_**o**_ and **S**_**θ**_ but do not explicitly estimate error in (**H**). Future applications could use alternative inventories, more process-resolved linear operators, or iterative adjoint-based optimization.

The formulation is not limited to national annual-average PM_2.5_ attainment. It can be adapted to other spatial scales, concentration targets, sectors, jurisdictions, and objectives, including exposure, health, equity, and multipollutant benefits. These outcomes should be evaluated in concentration or exposure space using an operator matched to the spatial, temporal, and chemical scale of the decision. Applying the framework to the 24-h PM_2.5_ standard, for example, would require a temporally resolved operator and a target based on daily design values or exceedance metrics.

### 4.2 Opportunities for Inverse Modeling to Inform Future Policy

Moving beyond NAAQS attainment, the inverse modeling framework suggests a more integrated approach to environmental policy design. Air quality, climate change mitigation, toxic exposures, and distributional inequality are often addressed in separate analytical silos, even when they arise from the same sources. A forward, brute-force search across all combinations of these objectives is computationally expensive and unlikely to identify the narrow intersections where multiple, multivariate benefits align. Because inverse modeling is grounded in optimization, it could offer a more tractable route to multiobjective design. Additional priorities (e.g., likelihood to reduce coemitted greenhouse-gas or toxic air contaminant concentrations, prioritizing mitigation in overburdened communities) could be embedded directly into the objective function or covariance structure. While no framework can eliminate the feasibility constraints and normative choices inherent in policy design, identifying where cobenefits are most achievable when regulatory capacity and political capital are limited may be as important as identifying the minimum pathway to attainment.

Viewed in this light, the present study advances air-quality policy design in three interconnected ways. First, it establishes an outcome-driven benchmark for compliance by estimating the minimum emissions intervention required to satisfy a regulatory threshold. This makes it possible to quantify the distance-to-compliance associated with nonoptimal or constrained strategies, even when those strategies ultimately achieve the same outcome. Second, by exploiting source-receptor relationships directly in the design step, the framework reveals obscured efficiencies across space, sectors, and precursor classes that are not apparent from emissions inventories or forward scenario testing alone. Third, the Bayesian formulation provides a foundation for uncertainty-aware design, making it possible to incorporate confidence, tolerance, and future policy priorities directly into the inference rather than treating attainment as a deterministic pass-fail exercise.

The broader implication is that concentration standards should not be viewed only as end points against which proposed plans are tested. They should also be treated as input constraints that reveal what the emissions system must do to comply. That shift from forward search to inverse formulation changes the role of atmospheric modeling in regulatory design. Instead of asking whether one candidate plan is better than another, it becomes possible to characterize the efficiency frontier itself and to quantify the additional burden imposed by policy choices that depart from the minimum-intervention benchmark. The outcome goes beyond a single prescriptive policy, building a transparent and data-constrained compliance roadmap of what to reduce, where to reduce it, and by how much.

## Supplementary Material



## Notes

Additional methodological details, sensitivity analyses, methodological considerations and limitations, and supplementary figures (PDF)
