# Supplementary material for: Inverse Modeling Identifies Efficient Emission Control Strategy for Mitigating PM2.5 Pollution
Source: Environ Sci Technol. 2026 Sep 29;60(40):28610–21. doi: 10.1021/acs.est.6c06581 (PMC13674066; doi:10.1021/acs.est.6c06581)
Supplement: Supplementary file 1 [file es6c06581_si_001.pdf]

Supporting Information for

Inverse modeling identifies efficient emission control strategy for mitigating $PM_{2.5}$ pollution

Chirag Manchanda[1*], Libby H. Koolik[1*], Alper Ünal[2,3], Inez Fung[4], Julian D. Marshall[3], Rachel Morello-Frosch[5,6], Alexander J. Turner[7], Robert A. Harley[1], Joshua S. Apte[1,5 **]

Affiliations:

[1] Department of Civil and Environmental Engineering, University of California, Berkeley, CA 94720, USA

[2] Eurasia Institute of Earth Sciences, Istanbul Technical University, Istanbul 34469, Turkey

[3] Department of Civil and Environmental Engineering, University of Washington, Seattle, WA 98195, USA

[4] Department of Earth and Planetary Sciences, University of California, Berkeley, CA 94720, USA

[5] School of Public Health, University of California, Berkeley, CA 94720, USA

[6] Department of Environmental Science, Policy, and Management, University of California, Berkeley, CA 94720, USA

[7] Department of Atmospheric Sciences, University of Washington, Seattle, WA 98195, USA

[*] Co-first author

[**] Corresponding author

Email apte@berkeley.edu

This PDF file includes 39 pages containing:

1. Supporting Text Sections S1-S5
2. Figures S1 to S21
3. SI References

Supporting Text

S1. Construction of the observation-informed baseline emissions state

S1.1. Baseline emissions processing and state-vector definition

The baseline emissions state was constructed from the 2022 National Emissions Inventory (NEI) and mapped to the InMAP Source-Receptor Matrix (ISRM) grid using SMOKE-analogous spatial-allocation procedures.[1–4] Emissions were resolved by grid cell, release layer, precursor pollutant, and sector, yielding a baseline emissions vector $\mathbf{x}_{\mathbf{NEI}} \in R^{n_e}$. The precursor dimension included primary $PM_{2.5}$, NO_x, NH_3, VOC, and SO_x, and the sector dimension included *Electricity, Food and Agriculture, Industrial, Residential, Transportation,* and *Other*. These sector categories are assigned based on the activities derived from thousands of individual Source Classification Codes (SCC). Electricity sources include all activities associated with electricity generation. Food and Agriculture includes agricultural machinery, commercial food processing, controlled burning for agricultural purposes, fertilizer use, livestock rearing, and tilling. Industrial sources include emissions from construction, solvents, industrial equipment, material production, oil and gas refining, and waste disposal. Residential sources include residential fuel combustion (e.g., natural gas heating homes), solvents, landscaping equipment, and waste disposal. Transportation sources are all emissions associated with aircraft, on-road vehicles (e.g., cars, trucks, buses), rail, and marine vessels. The “other” category captures all other anthropogenic emissions. Because this category combines remaining anthropogenic sources not assigned to the named sectors, we interpret “other” as a residual grouping rather than as a single coherent policy sector.

Sources that contribute to ambient $PM_{2.5}$ but are not treated as policy-adjustable in the attainment inversion were retained in the emissions state as an additional *Uncontrollable* category. These emissions are from wildfires, soil NO_x, biogenic VOC, and other non-anthropogenic contributions.[5–7] For less than ~1% of exceedance grid cells, uncontrollable emissions account for greater than ~50% of the concentrations at the baseline. Sources that are not included in the 2022 NEI (e.g., lightning) are not included in our analysis.

This construction serves two purposes. First, it preserves a single internally consistent emissions representation for both the forward and inverse calculations. Second, it allows uncontrollable sources to contribute to the modeled baseline concentration field while remaining fixed during the attainment optimization. Throughout the paper, the ISRM is used as the linear forward operator.[1–3] In this study, “uncontrollable” refers to emissions that contribute to the modeled baseline concentration field but are excluded from the admissible control vector during the attainment inversion. This classification is a modeling choice for the present proof-of-concept application and does not imply that all such sources are equally uncontrollable in every policy context.

$$y = \mathbf{H}x \qquad (S1)$$

where $y \in R^{n_r}$ is the vector of annual-average $PM_{2.5}$ concentrations across receptors, $x \in R^{n_e}$ is the emissions state vector, and $\mathbf{H} \in R^{n_r \times n_e}$ is the source-receptor transfer matrix. Although $PM_{2.5}$ formation includes nonlinear chemistry and transport, the ISRM provides a first-order linear approximation that makes both the baseline adjustment and the attainment inversion computationally tractable at continental scale.[1–3,8,9]

S1.2. Satellite-informed correction of the NEI baseline

Because the raw NEI inventory does not necessarily produce a baseline concentration field consistent with the observed spatial pattern of $PM_{2.5}$ exceedance, we constructed an observation-informed baseline state by assimilating a satellite-derived, model-assisted $PM_{2.5}$ surface for 2022 from the van Donkelaar *et. al*.[6] Let $\mathbf{y_{sat}}$ denote that concentration field, and let $\boldsymbol{\Sigma}_{\mathbf{sat}}$ denote the corresponding receptor-level uncertainty reported with the dataset. We then estimated an adjusted baseline emissions field $\mathbf{x_a}$ by solving a Bayesian update around the NEI prior.[6,10,11]

For this baseline-correction step, emissions were parameterized in log space as

$$x = x_{NEI} \odot exp(\varphi) \qquad (S2)$$

where φ is an unconstrained adjustment vector that allows the baseline inventory to move either upward or downward relative to the raw NEI. The corrected baseline was then obtained as the minimizer of

$$J(\varphi) = \frac{1}{2}\left(y_{sat} - \mathbf{H}\left(x_{NEI} \odot exp(\varphi)\right)\right)^T \mathbf{S_{o,base}^{-1}} \left(y_{sat} - \mathbf{H}\left(x_{NEI} \odot exp(\varphi)\right)\right) + \frac{1}{2}\,\varphi^T\,\mathbf{S_{\varphi}^{-1}}\varphi \qquad (S3)$$

The first term penalizes mismatch between the modeled $PM_{2.5}$ field and the satellite-informed baseline, and the second penalizes large departures from the NEI prior. This baseline adjustment is not intended as a formal source-attribution inversion. Rather, its purpose is to produce a baseline concentration and emissions state that is more representative of the observed 2022 $PM_{2.5}$ landscape and therefore more appropriate as the reference point for the subsequent attainment design.[6,10,11] The resulting observation-informed baseline underlies the baseline concentration and emissions patterns shown in main-text **Fig. 2** and in **Figs. S1-S6**.

S1.3. Receptor-space uncertainty in the baseline correction

The receptor-space covariance used in the baseline-correction problem combines observational uncertainty from the satellite product with structural model error in the ISRM. Specifically,

$$\mathbf{S_{o,base}} = \Sigma_{sat} + \Sigma_{ISRM} \qquad (S4)$$

where Σ_{sat} is a diagonal matrix containing the reported uncertainty of the satellite-derived $PM_{2.5}$ product at each receptor,[6] and Σ_{ISRM} is a diagonal matrix containing receptor-level ISRM model-error variances derived from the evaluation framework reported by Goodkind *et al.*[2] In this way, the baseline correction becomes conservative wherever either the observational product is uncertain or the reduced-complexity model is known to be less reliable.[3,6]

This combined covariance is central to the interpretation of the corrected baseline. The optimization is not being forced to reproduce the satellite surface exactly everywhere. Instead, it is being asked to reconcile the NEI with an observationally informed concentration field to the extent justified by both the measurement uncertainty and the model's own structural uncertainty. The resulting posterior x_a and $y_a = \mathbf{H}x_a$ are therefore best interpreted as an observation-informed baseline state rather than a fully unconstrained observation-matching solution. This baseline state is not intended to eliminate all emissions uncertainty. Rather, it provides a single internally consistent emissions and concentration reference state for the attainment inversion. Alternative inventories or alternative baseline-correction assumptions could change the detailed location, sector, or precursor mixture of the inverse solution, and can be evaluated by repeating the same workflow with alternative prior emissions fields. The effect of this correction on the emissions baseline is illustrated in **Figs. S4-S5**, while the resulting corrected baseline structure is

summarized in **Figs. S1-S3** and **Fig. S6**. The baseline correction changes both the magnitude and spatial distribution of the prior emissions field used by the attainment inversion. **Fig. S5** therefore reports both absolute and percent changes in emissions flux. Absolute-difference panels identify where the largest corrections occur in mass terms, whereas percent-change panels identify where the correction is large relative to the initial emissions estimate. Because percent changes are sensitive to small denominators, percent-change panels are masked in cells with negligible initial emissions.

S1.4. Prior covariance for the baseline-correction problem

The prior covariance $\mathbf{S}_{\boldsymbol{\varphi}}$ controls how freely the baseline inventory is allowed to deviate from the raw NEI. We parameterized $\mathbf{S}_{\boldsymbol{\varphi}}$ using the same tensor structure used later for the attainment inversion, but without the monotonic reduction constraint. In practice, this means that the baseline-correction problem allows both increases and decreases in emissions, while preserving the same dimensional organization across grid cell, release layer, precursor, and sector.

To maintain computational tractability, we represent the prior covariance in separable Kronecker form:

$$\mathbf{S}_{\boldsymbol{\varphi}} = \mathbf{K}_{\mathbf{g,base}} \otimes \mathbf{K}_{\mathbf{l,base}} \otimes \mathbf{K}_{\mathbf{p,base}} \otimes \mathbf{K}_{\mathbf{s,base}} \tag{S5}$$

where $\mathbf{K}_{\mathbf{g,base}}$, $\mathbf{K}_{\mathbf{l,base}}$, $\mathbf{K}_{\mathbf{p,base}}$, $\mathbf{K}_{\mathbf{s,base}}$ describe the marginal covariance structure across grid cells, release layers, precursor species, and sectors, respectively. In the present implementation, the covariance structure is designed so that, for a given grid cell and sector, all associated precursor emissions across all release layers are encouraged to move proportionally. In other words, the dominant coupling is within each grid-cell-by-sector activity block, rather than across distant locations. This reflects an activity-based control assumption: if activity in a given sector changes at a given location, then the full associated emissions mixture tends to increase or decrease together, rather than allowing individual precursors or layers to vary independently. This use of a common covariance architecture across both the baseline-correction and attainment problems ensures that the observation-informed baseline and the subsequent control design are embedded in a consistent inverse-model framework.[10,12]

This use of a common covariance architecture across the baseline-correction and attainment problems links the observation-informed baseline to the attainment inversion described in Sections S2.4-S2.5.

S2. Inverse attainment formulation and covariance structure

S2.1. Log-space attainment inversion

The attainment problem is solved relative to the corrected baseline state x_a. We define a log-space control variable θ such that

$$x = x_a \odot exp(\theta) \tag{S6}$$

with the elementwise constraint $\theta \leq 0$. This parameterization guarantees that all policy controls are represented as multiplicative reductions relative to the observation-informed baseline. A value θ_j leaves emissions component j unchanged, whereas increasingly negative values correspond to progressively larger reductions, up to complete removal of that baseline component.[10,12]

The inverse solution at each iteration is obtained by minimizing

$$J(\theta) = \frac{1}{2}\Big(y_o - \mathbf{H}\big(x_a \odot exp(\theta)\big)\Big)^T \mathbf{S_o^{-1}} \Big(y_o - \mathbf{H}\big(x_a \odot exp(\theta)\big)\Big) + \frac{1}{2}\,\theta^T\,\mathbf{S_\theta^{-1}}\theta \qquad (S7)$$

where y_o is the current attainment target, $\mathbf{S_o}$ is the receptor-space covariance governing tolerance to deviations from that target, and $\mathbf{S_\theta}$ is the prior covariance on emissions controls. The first term penalizes residual exceedance of the concentration target, while the second penalizes increasingly large deviations from the baseline emissions state.

S2.2. Flexible attainment target

Attainment was imposed using an iterative flexible target rather than a rigid equality constraint. At iteration *k*, the target field was constructed from the current concentration field ${y_o}^{k-1}$ as

$${y_o}^{k} = min\big({y_o}^{k-1}, y_{target}\big) \qquad (S8)$$

where y_{target} is the target $PM_{2.5}$ concentration and the minimum is taken elementwise. Receptors currently above the standard are capped at the standard, while receptors already below the standard are left unconstrained. The posterior concentration field from one iteration then becomes the prior concentration field for the next iteration.

This construction ensures that the inversion solves the actual regulatory problem, namely eliminating exceedances, without being penalized for concentrations that fall below the standard as a consequence of coupled emissions reductions. By contrast, a hard equality-style target penalizes additional reductions once a receptor falls below 9 $\mu g/m^3$, even when those same reductions are needed to bring nearby, more strongly violating receptors into attainment. As a result, the hard formulation can create artificial competition among neighboring exceedances and leave a subset of clustered receptors unresolved. The logic of this update is shown schematically in **Fig. S7**, and the resulting difference in concentration outcomes between the flexible and hard target formulations is shown in **Fig. S8**.

S2.3. Receptor-space covariance for the attainment inversion

Because the attainment target is a regulatory concentration threshold rather than an observed concentration field, the receptor-space covariance $\mathbf{S_o}$ in the attainment inversion reflects model structural uncertainty rather than observational uncertainty. Following the ISRM evaluation in Goodkind *et al.*[2], we constructed $\mathbf{S_o}$ as a diagonal matrix whose entries represent receptor-level $PM_{2.5}$ model-error variances:

$$\mathbf{S_o} = \Sigma_{ISRM} \qquad (S9)$$

This choice serves an important regularizing role. It prevents the inversion from prescribing unrealistically large reductions simply to force exact compliance at receptors where the modeled source-receptor response is comparatively uncertain. In other words, the target itself is deterministic, but the model used to translate emissions reductions into receptor-space improvements is not exact. Incorporating Σ_{ISRM} therefore makes the attainment solution conditional on what the reduced-complexity model can support defensibly.[10,12,13]

Operationally, this also ensures consistency between the baseline-correction problem and the attainment problem: the same characterization of ISRM error informs both the construction of the corrected baseline and the subsequent calculation of the minimum-intervention control pathway.

S2.4. Kronecker-structured prior covariance for emissions controls

The emissions-space covariance $\mathbf{S}_{\boldsymbol{\Theta}}$ governs how readily different parts of the emissions system can be adjusted away from baseline. Because the state vector is indexed by grid cell *g*, release layer *l*, precursor pollutant *p*, and sector *s*, a fully dense covariance over $\boldsymbol{\Theta}_{\mathbf{g,l,p,s}}$ would be computationally intractable. We therefore represent the prior covariance in separable Kronecker form.[10,12]

Let the unconstrained control covariance be

$$\mathbf{S}_{\boldsymbol{\Theta}}{}' = \mathbf{K}_{\mathbf{g}} \otimes \mathbf{K}_{\mathbf{l}} \otimes \mathbf{K}_{\mathbf{p}} \otimes \mathbf{K}_{\mathbf{s}} \qquad (S10)$$

where $\mathbf{K}_{\mathbf{g}}, \mathbf{K}_{\mathbf{l}}, \mathbf{K}_{\mathbf{p}}$ & $\mathbf{K}_{\mathbf{s}}$ describe the marginal covariance structure across grid cells, release layers, precursor species, and sectors, respectively. This decomposition makes it possible to encode structured policy assumptions while preserving computational tractability.

In the present application, the dominant coupling is not across distant locations, but within each local sector-activity block. Accordingly, the principal off-diagonal structure resides in the precursor and sector dimensions, whereas the grid and release-layer components are treated more simply. In particular, the precursor block $\mathbf{K}_{\mathbf{p}}$ is used to couple co-emitted species within a given location-sector combination, and the sector block $\mathbf{K}_{\mathbf{s}}$ allows sector-specific differences in control flexibility or penalty. This construction operationalizes the activity-based interpretation of the controls: if a given activity is reduced at a given location, then the associated precursor emissions are encouraged to decline together, rather than being adjusted independently one pollutant at a time.The same covariance structure can be used to encode differences in control feasibility or implementation cost. Larger penalties can be assigned to sectors, precursors, or locations where reductions are less feasible, while lower penalties can be assigned where controls are more readily available. Thus, $\mathbf{S}_{\boldsymbol{\Theta}}{}'$ should be interpreted not only as a regularization term, but also as a representation of the admissible control geometry.

More generally, $\mathbf{S}_{\boldsymbol{\Theta}}{}'$ should not be interpreted as a claim about the true statistical covariance of emissions. Rather, it defines the geometry of admissible control actions in the inverse problem. It determines which types of emissions updates are considered nearby in policy space and which are considered implausibly fragmented or costly. In the present implementation, $\mathbf{K}_{\mathbf{g}}$ and $\mathbf{K}_{\mathbf{l}}$ were taken to be identity matrices, while $\mathbf{K}_{\mathbf{p}}$ and $\mathbf{K}_{\mathbf{s}}$ carried the nontrivial coupling structure.

S2.5. Encoding controllable versus uncontrollable emissions through the covariance

Rather than removing uncontrollable sources from the state vector, we retain them in x and encode controllability directly in the prior covariance. Let $\mathbf{D}_{\mathbf{C}}$ be a diagonal controllability mask with entries 1 if component is controllable, and 0 otherwise. We then define the attainment-control covariance as

$$\mathbf{S}_{\boldsymbol{\Theta}} = \mathbf{D}_{\mathbf{C}}\, \mathbf{S}_{\boldsymbol{\Theta}}{}' \mathbf{D}_{\mathbf{C}} \; + \; \varepsilon(\mathbf{I} - \mathbf{D}_{\mathbf{C}}) \qquad (S11)$$

with ε chosen to be vanishingly small in practice. In the limit $\varepsilon \rightarrow 0$, uncontrollable components have zero prior variance in control space and are therefore fixed at baseline. They continue to contribute to $\mathbf{H}x$, but they are removed from the admissible control subspace. This is the mechanism by which wildfire-related and other uncontrollable sources shape the baseline and the attainment floor without being allowed to absorb any of the modeled policy burden.[5,6,14]

The same masking framework can be applied to any sector, precursor, location, or source class considered unavailable for control in a particular policy application. For example, an agriculture-constrained scenario could set the corresponding entries of $\mathbf{D_C}$ to zero, impose a maximum reduction fraction, or assign those entries a larger prior penalty. The present analysis uses a broad controllable-anthropogenic mask to demonstrate the framework, but more detailed applications could encode available control technologies or jurisdiction-specific regulatory authority directly in $\mathbf{D_C}$ and $\mathbf{S_\theta}$. For the no-agricultural-control sensitivity presented in Fig. S21, entries corresponding to agricultural emissions were set to zero in $\mathbf{D_C}$, holding that sector at its baseline emissions while allowing all other modeled-controllable sectors to vary.

This implementation has both conceptual and computational advantages. Conceptually, it preserves a single full-state representation of the emissions system. Computationally, it allows controllability to be imposed cleanly within the same Kronecker-based covariance framework used for the rest of the inversion. The practical consequences of this distinction are shown in **Fig. S1**, which quantifies the contribution of uncontrollable emissions to baseline concentrations and exceedance.

S2.6. Rationale for the quadratic norm in a universal-attainment framework

The attainment inversion in Eq. (S7) uses a quadratic receptor-space misfit,

$$\frac{1}{2}\Big(y_o - \mathbf{H}\big(x_a \odot exp(\theta)\big)\Big)^T \mathbf{S_o^{-1}} \Big(y_o - \mathbf{H}\big(x_a \odot exp(\theta)\big)\Big)$$

rather than an L1 norm or another robust alternative. This choice is tied to the policy question posed here. We do not formulate attainment as a problem of maximizing aggregate compliance benefit under a fixed control budget, nor as a problem of improving as many receptors as possible while potentially leaving a small number unresolved. Instead, we frame attainment as a universal threshold problem: the objective is to identify a minimum-intervention control allocation that can bring the modeled receptor field into compliance, subject to uncertainty, controllability, and the admissible control structure encoded by the covariance terms.

Under this objective, the quadratic norm has an important interpretation. Because the penalty grows more rapidly with residual magnitude, it increasingly disfavors solutions that leave a small number of receptors with large remaining exceedances while improving many others only modestly. In that sense, the L2 norm is well aligned with a universal-attainment framing, where even a few unresolved exceedances remain policy-relevant. By contrast, an L1 norm penalizes residuals linearly and therefore places less additional weight on large remaining exceedances. In practice, this makes L1 more naturally aligned with a different planning problem: maximizing aggregate progress, minimizing total absolute mismatch, or achieving the greatest partial compliance under a constrained intervention budget, even if a subset of difficult receptors remains above the standard.

This distinction is especially important for annual-average $PM_{2.5}$, where the regulatory objective is threshold attainment rather than minimizing concentration everywhere in an average sense. In such a setting, a formulation based on L1 residual minimization could favor solutions that distribute reductions efficiently in aggregate while tolerating a limited number of persistent exceedances if eliminating them is comparatively costly. That is a reasonable objective for a budget-limited or partial-compliance optimization problem, but it is not the policy question considered here. Our question is instead: what is the minimum-intervention allocation that can bring the entire modeled receptor field into attainment, once flexible target construction, model uncertainty, and controllability constraints are taken into account?

The use of a quadratic norm is also consistent with the Bayesian inverse structure adopted here. In the MAP formulation, the quadratic receptor-space term arises naturally from a Gaussian approximation to uncertainty and allows $\mathbf{S_o}$ to act as an explicit weighting on the relative penalty assigned to residual exceedances at different receptors. This is important in the present application because robustness to receptor-specific model uncertainty is already handled directly through $\mathbf{S_o}$. Receptors where the reduced-complexity source-receptor response is less certain are downweighted in a physically interpretable manner, reducing the incentive to prescribe extreme controls simply to force exact compliance at locations where the modeled response is less defensible. Replacing the quadratic norm with an unweighted L1 norm would therefore not simply provide a more robust version of the same attainment problem; it would redefine the objective while discarding the natural covariance-weighted interpretation already built into the inverse.

A further practical consideration is computational. The present formulation combines a smooth quadratic objective in receptor space with a log-space parameterization of emissions controls and structured covariance operators in emissions space. This yields a differentiable inverse problem that can be solved efficiently using the iterative linearized strategy described above. An L1 formulation would introduce a non-smooth residual term and would generally require auxiliary-variable, sub gradient, or other large-scale non-smooth optimization methods. Such a formulation is possible in principle, but it would define a different planning problem and would be substantially less natural within the covariance-weighted inverse architecture used here.

We therefore do not view L2 as universally superior to L1. Rather, we view it as the appropriate choice for the specific regulatory framing of this study. The present goal is to identify a minimum-intervention benchmark for domain-wide attainment, not to maximize partial compliance or average improvement. Under that framing, the quadratic norm, flexible attainment target, and covariance-weighted inverse structure together define a coherent formulation of the universal $PM_{2.5}$ NAAQS attainment problem.

S3. Reference solution pathways and forward ensemble analyses

S3.1. Forward solution-space ensemble

To characterize the broader forward design space shown in **Fig. 3**, we generated a one-million-member ensemble of emissions-control scenarios over the controllable anthropogenic system. Each scenario was defined in terms of grid-cell-by-sector activity controls, with a reduction fraction $u_{g,s} \in [0,1]$ assigned to each grid-cell-by-sector pair. That activity reduction was then propagated proportionally to all precursor and release-layer components within the corresponding location-sector combination:

$$\Delta x_{g,l,p,s} = u_{g,s} \times x_{a\ (g,l,p,s)} \qquad (S12)$$

This forward-control geometry mirrors the same activity-based coupling embedded in the attainment inversion. Reduction fractions were generated using Latin Hypercube Sampling and discretized in 1% increments from 0 to 100%. The resulting emissions field for each scenario was evaluated with the ISRM to obtain the corresponding $PM_{2.5}$ concentration surface and summary attainment statistics.[1,3,15–17]

We evaluated the sensitivity of the sampled outcome space to ensemble size using nested subsets containing 0.125, 0.25, 0.50, and 1.00 million simulations and compared their outcome envelopes with the boundary obtained from two million simulations (**Fig. S9**). Coverage expanded substantially from 0.125 to 0.5 million simulations, particularly at moderate emission reductions, but the incremental expansion diminished thereafter. Most importantly, the one-million-simulation envelope closely traces the two-million-simulation boundary across most of the outcome space, indicating that one million simulations were sufficient to capture the principal range and shape of the full solution space.

Importantly, this ensemble was not constructed to approximate the posterior distribution of the inverse problem. The sampled scenarios were generated independently of the inverse objective function, covariance weighting, and flexible attainment updates; they are simply randomized control allocations evaluated through the same forward operator **H**. The purpose of this ensemble is therefore descriptive rather than inferential: not to sample the inverse posterior, but to provide an empirical map of feasible outcomes under many plausible control allocations.

The purpose of this ensemble is not to exhaustively enumerate the full design space, which is combinatorially impossible, but to provide an empirical map of feasible outcomes under many plausible control allocations. The broad spread of the resulting cloud in **Fig. 3** shows that aggregate emissions reduction alone is not a sufficient predictor of attainment; the placement of reductions across space and sectors matters strongly. The inverse solution is then overlaid on this cloud as a benchmark for minimum-intervention attainment. The spatial allocation patterns associated with the inverse, Least Efficient, Local, and Maximalist pathways are shown in **Figs. S10-S12**. **Figs. S10-S12** show both absolute emissions-flux reductions and percent reductions relative to the corresponding baseline emissions field. Some reductions in these spatial allocation maps appear over ocean or near-shore grid cells. These reductions do not represent controls on natural oceanic background emissions but instead correspond to anthropogenic emissions represented in over-water or near-shore inventory grid cells.

S3.2. Maximalist solution pathway

In addition to the inverse solution, we constructed a sequential "Maximalist Solution" pathway, also referred to as the "greedy approach" in optimization literature,[18] to illustrate how a locally rational control strategy performs relative to the globally optimized benchmark. Starting from the corrected baseline, the "Maximalist" algorithm repeatedly identifies the next candidate activity reduction that yields the largest immediate improvement in the attainment objective per unit control effort. That control increment is applied, the concentration field is recomputed, and the remaining candidates are re-ranked. This process continues until all modeled exceedances are removed.

Because the Maximalist strategy selects controls one step at a time, it captures the most obvious marginal gains first but does not account for how early decisions reshape the structure of the remaining problem. As the residual exceedances become concentrated in a smaller set of harder-to-control receptors, the pathway typically requires progressively broader and less efficient reductions. This is why the "Maximalist Solution" in **Fig. 3** reaches attainment only after substantially larger system-wide control effort than the inverse solution. The value of this pathway is interpretive: it shows that local marginal effectiveness alone is not sufficient to recover the global minimum-intervention pathway, even when each individual decision is rational in isolation.[15–19] This behavior is reflected in main-text **Fig. 3** and in **Fig. S12**, which shows the broader and more diffuse reduction pattern required by the Maximalist pathway.

S3.3. State-wise reallocated solution pathway

To evaluate to what extent the inverse model's recommendations must be implemented at the grid scale's precision, we perform a state-wise reallocation scenario. This scenario preserves the same total sector-specific reductions in each state as the original inverse solution but redistributes those reductions uniformly across all relevant grid cells within the state. Meaning, if the sum of an individual state's reductions to transportation-related emissions is 35%, we uniformly reduce transportation-related emissions within each grid cell by 35%. We do not rerun the inverse model on a coarser grid or test whether the solution is invariant to grid definition. Instead, we deliberately aggregate the grid-resolved inverse solution to state-sector totals and then reapply those reductions across the original grid, removing the within-state spatial specificity of the solution while preserving its state-sector structure. This analysis is therefore not a sensitivity test of the

model's performance on high resolution grids, but instead a sensitivity test of how imprecise implementation performs when compared to population exposures.

As shown in **Fig. S13**, this state-wise reallocation captures a substantial fraction of the structure of the inverse solution, particularly through the center of the population exposure distribution, but it is less efficient in the upper tail where attainment is determined. The difference between the grid-resolved and state-wise reallocated pathways therefore reflects the cost of losing within-state source-location information, rather than the effect of changing the underlying model resolution. This result is useful for interpretation. It shows that the fully grid-resolved inverse solution provides the most efficient benchmark, but also that the state-and-sector structure of the solution already contains a considerable share of the policy-relevant information. In that sense, the grid-resolved inverse solution should be interpreted as a reference benchmark for minimum-intervention attainment, while the state-wise decomposition provides a more aggregated and policy-interpretable starting point for subsequent forward evaluation. This provides a useful bridge between the fully grid-resolved benchmark and the more aggregated state-and-sector interpretation emphasized in **Fig. 4**.

More generally, the grid-resolved benchmark should be interpreted at the spatial support of the source-receptor operator. Each grid cell represents an aggregate of sources within that cell, so a more detailed operator could redistribute reductions within large or heterogeneous cells. However, the variable-resolution ISRM grid already provides finer spatial support in more populated areas, where exposure gradients and attainment-relevant concentration changes are most consequential. Thus, **Fig. S13** should be interpreted as a test of spatial aggregation from grid to state-sector summaries, not as a test of grid invariance.

S4. Response-based $PM_{2.5}$-equivalent metric

Several analyses in the main text compare mitigation effort and solution structure across sectors and pathways that differ in $PM_{2.5}$ precursor composition. Because the ISRM is precursor-specific, emissions mass is not directly comparable across precursor classes. A unit reduction in NH_3, for example, is not commensurate with a unit reduction in primary $PM_{2.5}$, NO_x, SO_x, or VOC in terms of its modeled $PM_{2.5}$ consequence. We therefore report aggregate emissions changes on a primary $PM_{2.5}$-equivalent basis for cross-sector and cross-pathway comparisons. This additivity is what enables the cumulative state-level decomposition in **Fig. 4** and the precursor-resolved decompositions in **Figs. S15-S19**.

This equivalence is used only as an accounting normalization for aggregation and visualization. All concentration calculations, attainment diagnostics, and inverse optimizations are performed in the native precursor-resolved emissions space using the full ISRM operators.[3]

S4.1. Defining precursor equivalence through a weighted concentration-response functional

Let $H^{(k)}$ denote the ISRM operator for precursor *k*, and let $e_j^{(k)}$ denote emissions of precursor *k* from source component *j*, where *j* indexes source location and release layer (and, where relevant, sector). The concentration contribution at receptor *i* from that source component is

$$C_i^{(k)} = H_{i,j}^{(k)} e_j^{(k)} \qquad (S13)$$

To compare emissions across precursors, we first define a scalar functional that maps any concentration field to a single summary response:

$$L(C) = \sum_i w_i C_i \qquad (S14)$$

where the weights w_i are nonnegative and normalized such that $\sum_i w_i = 1$. In the present work, we use population-weighted receptor averaging, so that $w_i = p_i \;\; / \;\; \sum_i p_i$

where p_i is the population attributable to receptor grid cell *i*. Under this definition, *L*(*C*) is the population-weighted average annual $PM_{2.5}$ response across the contiguous United States. This choice provides a neutral, receptor-based reporting scale that is appropriate for a paper focused on attainment of an ambient concentration standard.

For a unit emission from source component *j* in precursor *k*, the corresponding scalar response is

$$R_j^{(k)} = \sum_i w_i H_{i,j}^{(k)} \qquad (S15)$$

Likewise, for a hypothetical unit emission of primary $PM_{2.5}$ from the same source component j, the corresponding response is

$$R_j^{(PM)} = \sum_i w_i H_{i,j}^{(PM)} \qquad (S16)$$

We then define the source-specific primary $PM_{2.5}$-equivalent scaling factor for precursor *k* at source component *j* as

$$\rho_j^{(k)} = R_j^{(k)} / \; R_j^{(PM)} \qquad (S17)$$

and the corresponding primary $PM_{2.5}$-equivalent emissions as

$$e_{PMeq,j}^{(k)} = \rho_j^{(k)} \times \; e_j^{(k)} \qquad (S18)$$

This definition has an exact interpretive property:

$$L\left(H_{i,j}^{(k)} e_j^{(k)}\right) = L\left(H_{i,j}^{(PM)} e_{PMeq,j}^{(k)}\right) \qquad (S19)$$

That is, $e_{PMeq,j}^{(k)}$ is exactly the amount of primary $PM_{2.5}$ emitted from the same source component j that would produce the same weighted $PM_{2.5}$ response under the chosen functional *L*. The equivalence is therefore defined directly from the same source-receptor model used everywhere else in the paper, rather than from a fixed precursor multiplier.

S4.2. Aggregate $PM_{2.5}$-equivalent emissions

Using the source-specific conversion above, the $PM_{2.5}$-equivalent emissions associated with any multi-precursor emissions field are given by

$$E_{PMeq} = \sum_k \sum_j \rho_j^{(k)} e_j^{(k)} \quad (S20)$$

Likewise, for any subset of sources G, such as a given state, sector, or pathway, the corresponding $PM_{2.5}$-equivalent total is

$$E_{PMeq}(G) = \sum_k \sum_{j \in G} \rho_j^{(k)} e_j^{(k)} \quad (S21)$$

Because this conversion is linear in emissions, the resulting $PM_{2.5}$-equivalent totals are exactly additive across states, sectors, and precursors. This makes the metric well suited for the state-level cartographic decompositions in **Fig. 4**, **Figs. S6,** and **Figs. S15–S19**.

S4.3. Interpretation and advantages of the source-specific conversion

This source-specific formulation has several advantages.

First, it is anchored directly to the ISRM and therefore remains fully consistent with the same atmospheric-response representation used for both the baseline calculations and the inverse design. The $PM_{2.5}$-equivalent accounting is not imposed externally; it is derived from the model itself.

Second, the conversion preserves the spatial and sectoral support of emissions. Each precursor emission is re-expressed as an equivalent amount of primary $PM_{2.5}$ emitted from the same source component j, rather than being redistributed across space or across sectors. This is important for interpretation of state-level and sector-level burdens.

Third, the conversion preserves source heterogeneity. Two equal mass reductions in the same precursor need not receive the same $PM_{2.5}$-equivalent weight if they occur in different places or release layers, because their source-receptor leverage can differ. In that sense, the metric reflects not only precursor identity, but also the location-specific and layer-specific influence of emissions within the modeled atmospheric system.

More broadly, this definition makes clear that $PM_{2.5}$-equivalent mass is not a conserved chemical quantity and should not be interpreted as one. It is a response-normalized measure of control effort, defined relative to the weighted $PM_{2.5}$ response functional L and the same linear source-receptor mapping used throughout the paper.

S5. Role of uncontrollable emissions in the attainment problem

Uncontrollable emissions enter the framework in two distinct but complementary ways. First, they contribute directly to the baseline $PM_{2.5}$ field through the forward operator and therefore shape the spatial pattern of nonattainment. Second, because their corresponding control variances are set effectively to zero in S_θ, they are excluded from the admissible control space. Their effect is therefore to define part of the irreducible background burden against which controllable anthropogenic reductions must operate.

This distinction is important for interpretation of both the baseline maps and the inverse-model solution. The presence of uncontrollable emissions means that not all exceedance is, even in principle, removable through the modeled policy levers. At the same time, **Fig. S1** shows that although uncontrollable sources are regionally important, especially in parts of the western United States, most exceedance grid cells remain

dominated by the controllable share of the system. The attainment problem is therefore not trivialized by uncontrollable burden, but it is constrained by it in a physically meaningful way.

Uncontrollable emissions therefore alter both the feasibility and the interpretation of the attainment problem. In locations where their contribution is large, the remaining controllable margin to reach the standard is correspondingly smaller, and in rare cases the standard may be exceeded even in the absence of any further controllable anthropogenic emissions reductions. The inverse solution should therefore be interpreted as the minimum controllable intervention required conditional on this fixed background, rather than as a claim that all nonattainment is fully removable within the modeled policy space. This framing is consistent with the role of our inverse model as a reference benchmark: it identifies the most efficient controllable pathway given the atmospheric and policy constraints represented in the model, while making explicit where irreducible background burden limits what controllable mitigation alone can achieve. Annual variability in uncontrollable emissions is especially important for wildfire-related $PM_{2.5}$. Because wildfire emissions vary substantially from year to year, the number and location of receptors that can be brought below the annual $PM_{2.5}$ standard through controllable anthropogenic reductions alone may also vary. In higher-wildfire years, the fixed background burden may increase the number of receptors that remain above the standard even after modeled controllable emissions are reduced. In lower-wildfire years, the anthropogenic reduction burden may be smaller. Future regulatory applications should therefore evaluate multiple emissions and meteorological years, or explicitly include wildfire scenarios, to distinguish persistent anthropogenic attainment challenges from year-specific background contributions.

S6. Methodological Considerations and Limitations

A key strength of the inverse modeling framework is that implementation constraints can be incorporated explicitly. The model can exclude certain sources, sectors, or locations from the control space or impose upper bounds on allowable reductions. Future applications could encode costs or policy preferences through the prior covariance structure or additional penalty terms to solve for the least-monetary cost, least-disruptive, or other multi-objective pathways (e.g., total population exposure reduction, monetized health benefits, distributional equity, environmental-justice communities, multi-pollutant benefits) or constraints that reflect available control technologies. In this sense, the present minimum-intervention benchmark is one of a broader class of inverse planning problems that could be tailored to different policy objectives. That flexibility matters because many $PM_{2.5}$-contributing sources also contribute to other forms of environmental burden, and therefore the most desirable strategy may not be the one that minimizes total emissions reduction.

The framework should therefore be viewed as a bridge between atmospheric source-receptor modeling and the broader attainment-planning literature. Existing regulatory analyses often evaluate control packages defined by available technologies, costs, jurisdictional authority, or benefit-cost objectives. By contrast, the inverse solution identifies the minimally viable aggregate emission reduction before imposing those additional implementation filters. This distinction allows the inverse model to serve as a benchmark: candidate strategies can be evaluated by how much additional emissions reduction, cost, or distributional tradeoff they require relative to the minimum-intervention benchmark.

A fundamental limitation lies in the transfer function. Our framework depends critically on the transfer function **H**, that maps emissions changes to concentration responses. In this study, we use the ISRM because it is computationally tractable, policy-relevant, and has been evaluated and applied extensively in the forward modeling literature for air quality and exposure analyses.[2,3,20–23] Reduced-complexity models such as the ISRM inevitably omit some aspects of atmospheric physics and chemistry, and may be less reliable in regions with complex terrain, strong nonlinearity in secondary aerosol formation, or source mixtures that are imperfectly represented in the underlying training simulations used to construct the operator. Inverse

model applications are sensitive to inaccuracies in **H**. Here, uncertainty in the target field and in the emissions prior is represented through $\mathbf{S_o}$ and $\mathbf{S_\theta}$ but error in **H** is not modeled explicitly. We partially address this issue by propagating concentration uncertainty into the optimization and de-emphasizing reductions in locations where the concentration target is more uncertain. This reduces sensitivity to uncertain target values, but it is not a substitute for a more accurate transfer operator. To our knowledge, this treatment differs from the more common use of InMAP/ISRM-style reduced-complexity models as deterministic screening tools for estimating exposure or health changes under candidate scenarios.

Other limitations of the ISRM are less straightforward to quantify through $\mathbf{S_o}$ and $\mathbf{S_\theta}$. For example, the ISRM provides annual-average concentration changes from annual-average emissions changes, and this neglects the independently time-variant sources and atmospheric processes that modulate and/or amplify each other. Similarly, there are well-documented biases in space and by precursor pollutants that should be carefully considered when interpreting results.[24] Altogether, the ISRM is best used for marginal, time-invariant changes (i.e., stable emissions changes that do not dramatically affect overall atmospheric chemistry), and therefore should not be used as the final arbiter of attainment for regulatory compliance, especially in non-attainment areas with strong seasonality. Future work could replace or augment the present operator with a more process-resolved linear transfer function while retaining the inverse formulation. Alternatively, the attainment problem could be reformulated as an iterative adjoint-based optimization, which would provide objective gradients rather than the explicit transfer matrix used here and require repeated forward-adjoint calculations. The main contribution of this work does not depend on any one specific transfer function, as the conceptual advance is the reframing of regulatory design as an inverse problem.

The emissions inventory is an additional source of uncertainty. In forward scenario analysis, inventory errors affect the predicted concentration response to a proposed control strategy. In inverse modeling, they also influence the admissible control space, because reductions can only be assigned to emissions represented in the baseline inventory. Thus, uncertainty in emissions magnitude, spatial allocation, and sector attribution can shift the detailed source-sector-location mixture of the minimum-intervention pathway. Future applications could propagate this uncertainty by repeating the inversion using multiple methodologically distinct emissions inventories (e.g., the NOAA's Fuel-Based Inventory for Vehicle Emissions, the European Commissions' Emissions Database for Global Atmospheric Research), spatial surrogates, or inventory perturbation ensembles.

A useful feature of our method is the portability of different inputs, spatial scales, and regulatory challenges. The same principle applies to future health or equity applications of this framework. Estimating distributional outcomes directly from emissions reductions would miss the spatially heterogeneous source-receptor relationships that determine exposure. The inverse framework can incorporate health or equity objective functions, but those objectives should be evaluated in concentration or exposure space, using a source-receptor operator appropriate to the spatial, temporal, and chemical scale of the decision. Thus, the appropriate model may range from a reduced-complexity operator for broad screening or national benchmarking to a process-resolved chemical transport model for regulatory applications where local gradients, nonlinear chemistry, or episodic meteorology are central. While we demonstrate the inverse modeling framework here for national annual-average $PM_{2.5}$ attainment, the formulation is not limited to this setting (in space, pollutant, or time scale). It could be applied at local, regional, or state scales; to different concentration targets; or to settings in which only a subset of sectors, sources, or jurisdictions are available for control. This flexibility also extends to the temporal form of the concentration target. Although we focus on annual-average attainment, the same logic could be applied to the 24-hour $PM_{2.5}$ standard using a temporally resolved source-receptor operator and a target function based on daily design values or exceedance metrics. Such an application would need to represent the episodic and seasonal processes that

can drive short-term $PM_{2.5}$ exceedances, including meteorology, secondary formation, residential or agricultural seasonality, wildfire smoke, and stagnation events.

Supporting Figures

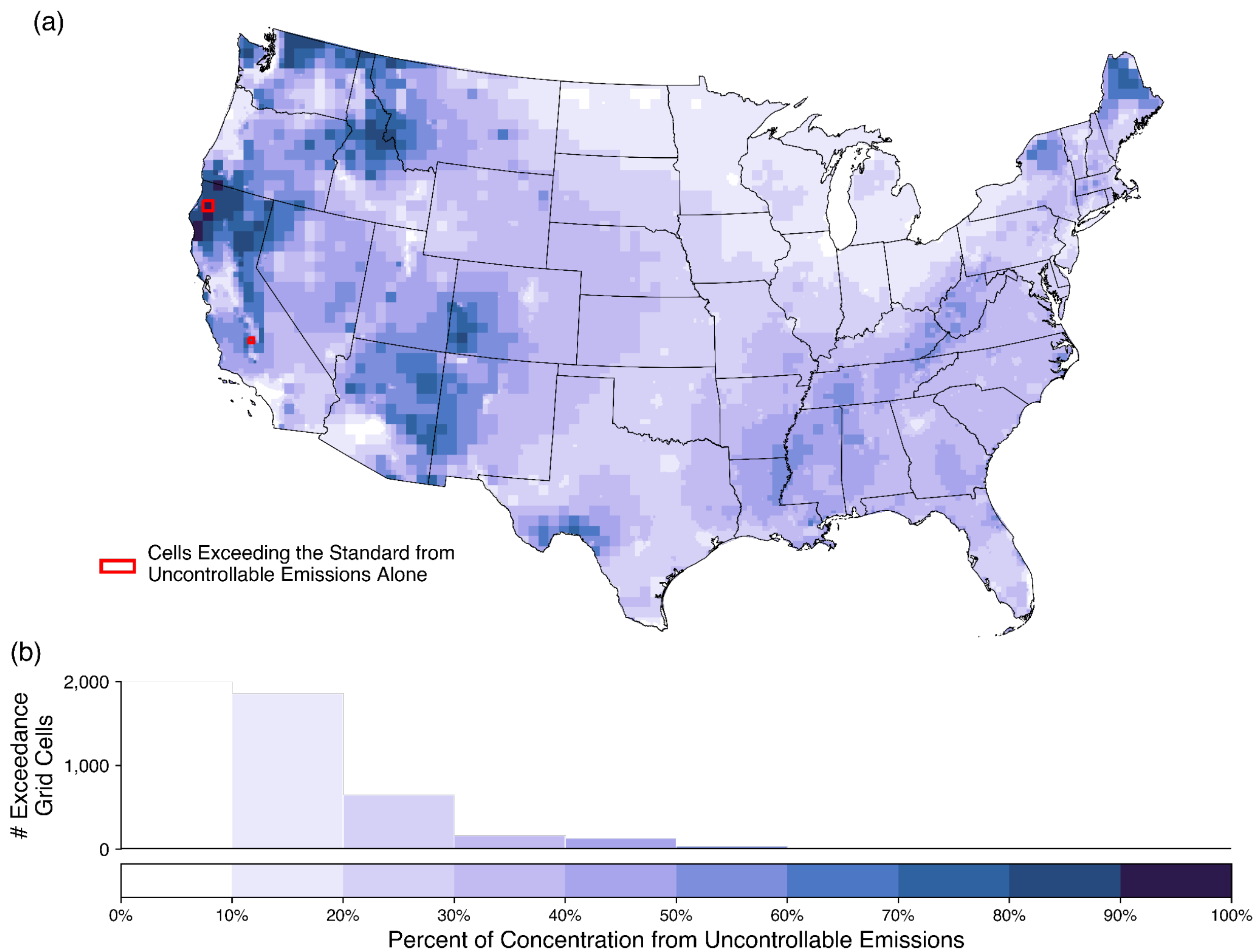

Fig. S1. Contributions of uncontrollable emissions to baseline concentrations.

Role of uncontrollable emissions in the baseline $PM_{2.5}$ concentration field. Panel **(a)** maps the fraction of modeled baseline concentration attributable to uncontrollable emissions at each grid cell, including sources such as biogenic VOC and wildfire-related contributions. Their influence is strongest across the western United States, especially along the Pacific Coast, Pacific Northwest, and parts of the interior West, with smaller but still non-negligible contributions across parts of the eastern U.S. Two grid cells, outlined in red in California, exceed the 9 $\mu g/m^3$ standard from uncontrollable emissions alone, indicating that a small number of receptors face an attainment floor that cannot be eliminated through the modeled anthropogenic controls. Panel **(b)** shows the distribution of uncontrollable contributions among grid cells that exceed the NAAQS in the baseline state. Most exceedance grid cells fall in the 10–30% uncontrollable contribution range, with progressively fewer cells at larger fractions and only a very small number above 50%. Taken together, these panels show that uncontrollable emissions are regionally important and can materially raise the baseline, especially in the West, but that most noncompliant grid cells remain dominated by the controllable share of the system. This justifies treating uncontrollable emissions as an irreducible background burden in the inverse design while still focusing the optimization on anthropogenic reductions.

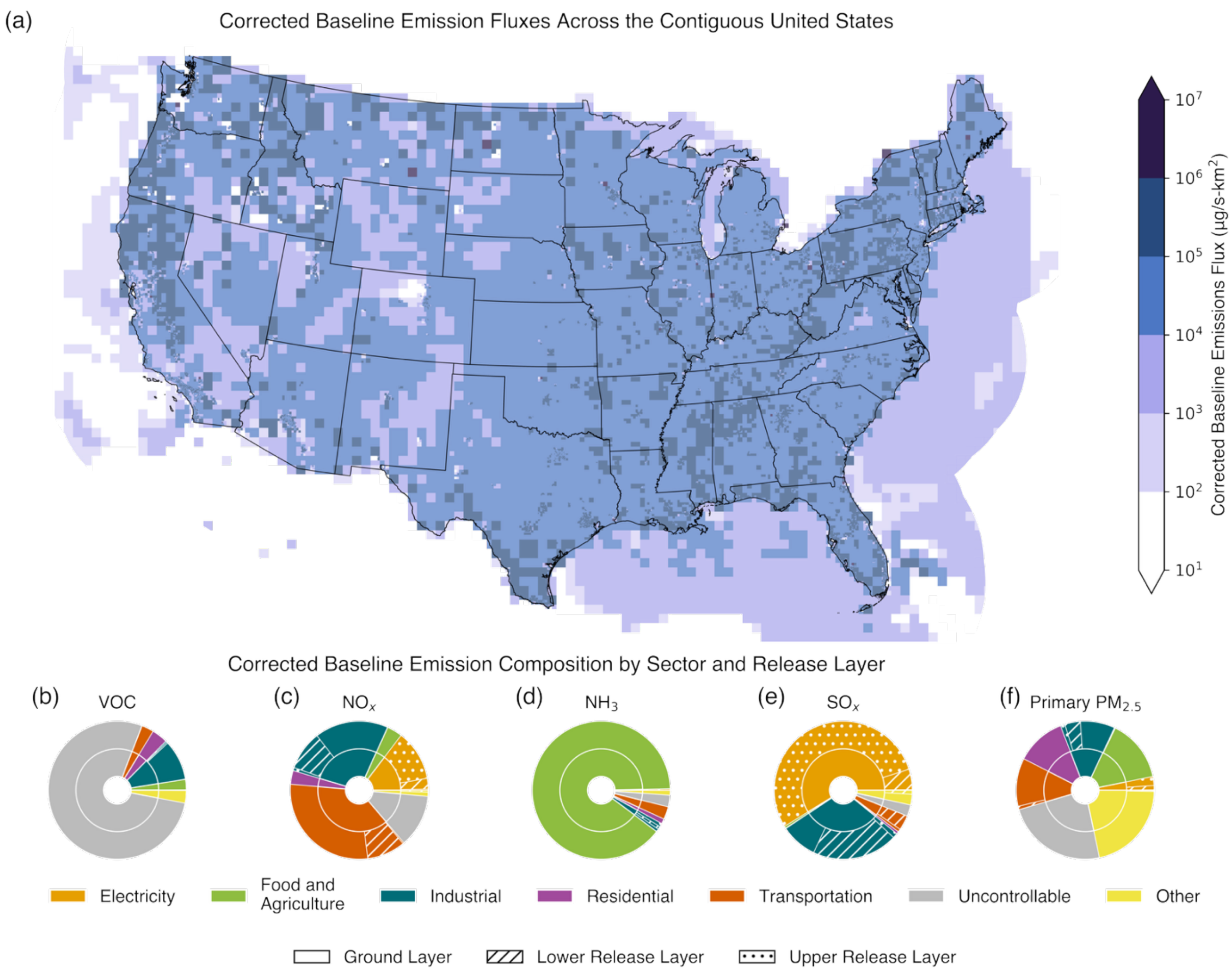

Fig. S2. Spatial and sectoral distribution of the baseline-corrected emissions inventory.

Baseline anthropogenic and uncontrollable emissions after observational correction of the 2022 inventory. Panel **(a)** maps total $PM_{2.5}$-equivalent emissions flux across the contiguous United States. Panels **(b-f)** decompose nationwide emissions for each precursor pollutant by economic sector and release layer. In each pie chart, the inner ring denotes sectoral composition and the outer ring denotes release layer. The corrected inventory concentrates large emissions in major metropolitan areas, industrial corridors, and agricultural regions. Sectoral composition differs substantially by precursor: VOC emissions are heavily influenced by uncontrollable sources, NH_3 is dominated by Food and Agriculture, SO_x is concentrated in electricity-generation and industrial sources, and NOx and primary $PM_{2.5}$ reflect more mixed sectoral contributions. With the exception of SO_x, most emissions are emitted from the ground layer.

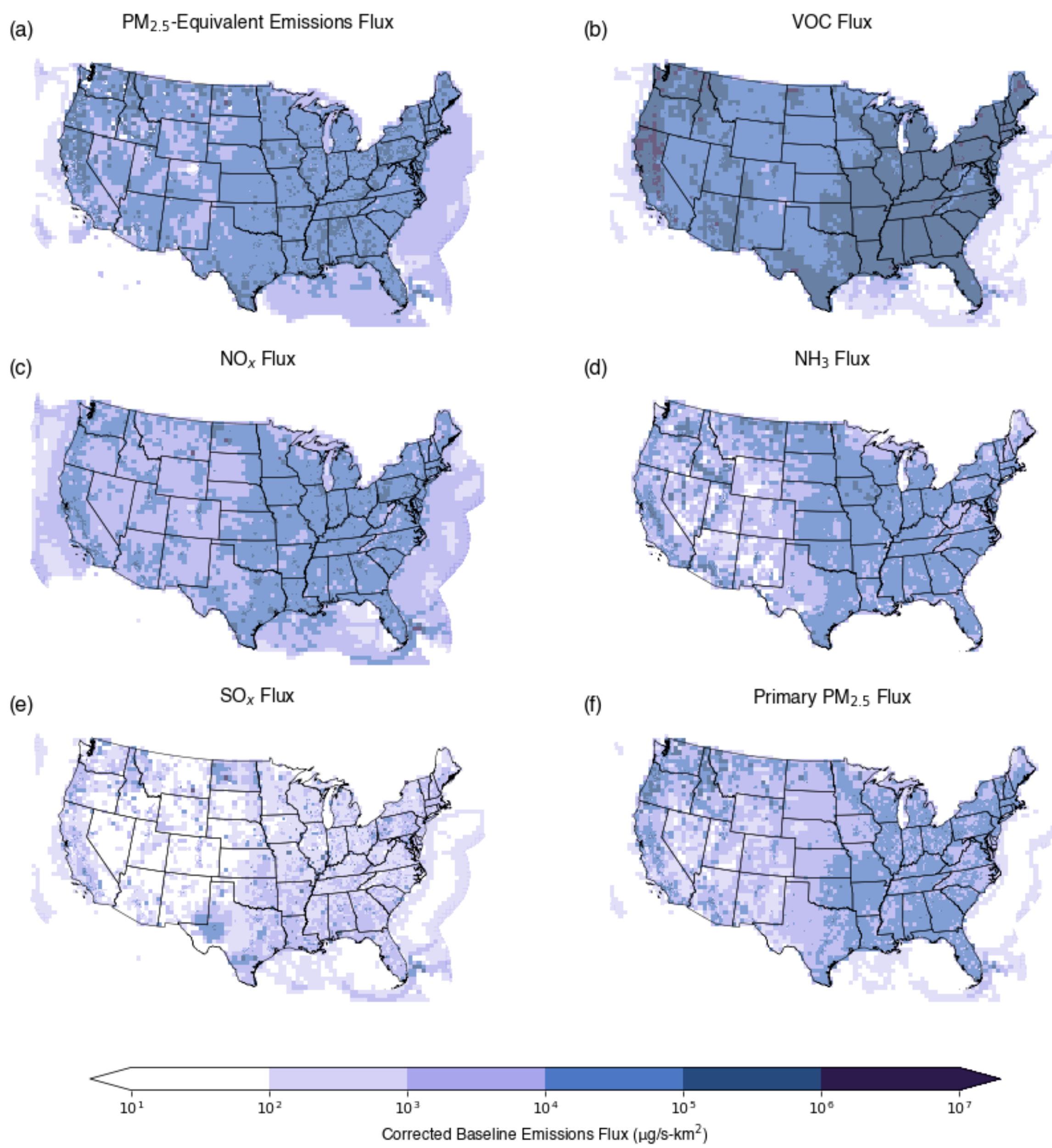

Fig. S3. Spatial distribution of each precursor pollutant in the baseline-corrected emissions inventory.

Spatial distribution of $PM_{2.5}$-equivalent and precursor-specific emissions fluxes in the baseline-corrected inventory. Panel **(a)** reproduces total PM-equivalent emissions, while panels **(b-f)** show VOC, NO_x, NH_3, SO_x, and primary $PM_{2.5}$, respectively. The precursor fields differ markedly in spatial structure: NO_x follows urbanization and transportation corridors more closely, NH_3 is concentrated in agricultural regions, SO_x is localized around power-generation and industrial sources, and VOC and primary $PM_{2.5}$ are more spatially diffuse. These contrasts motivate the precursor-resolved formulation used in the inverse model.

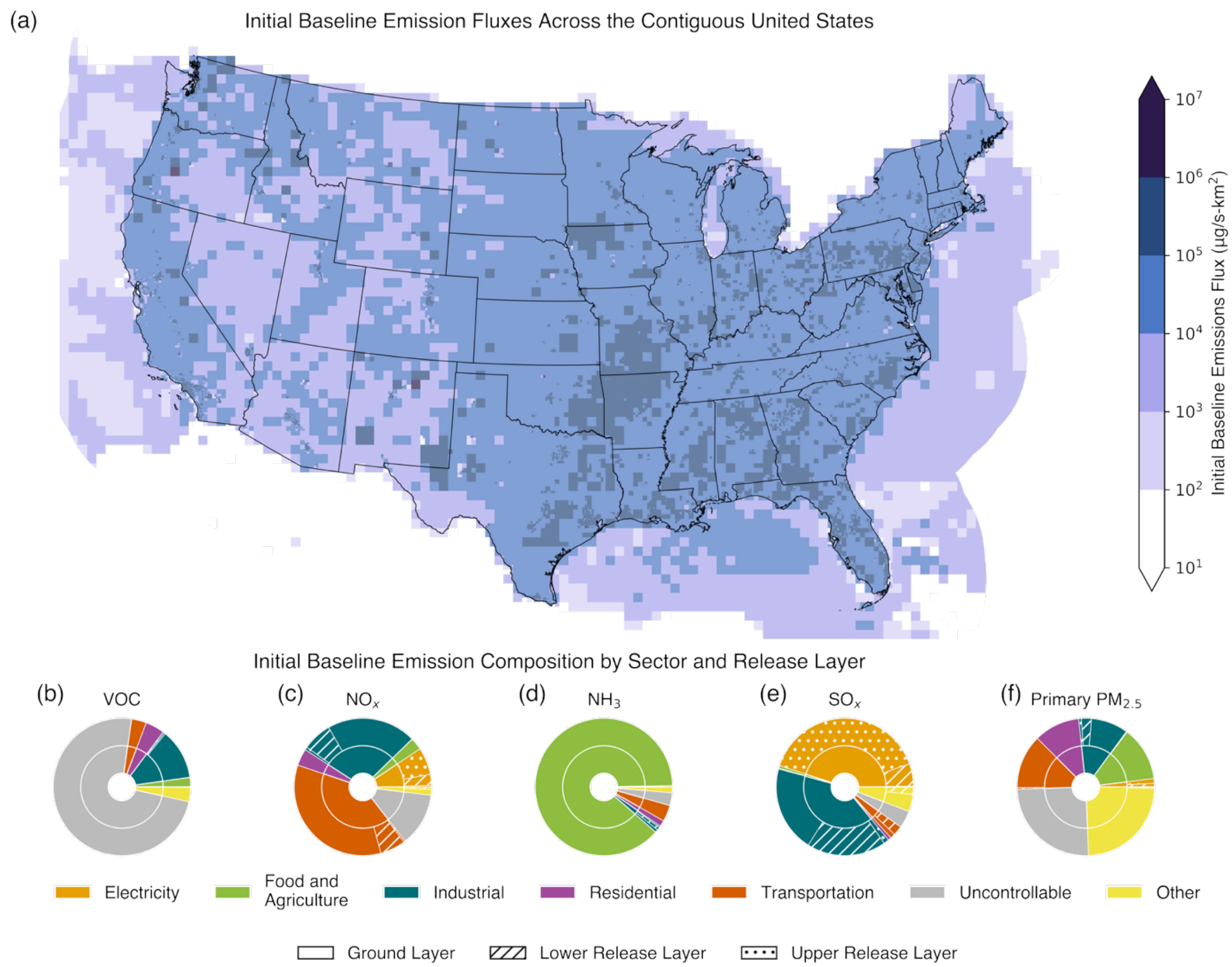

Fig. S4. Spatial and sectoral distribution of the initial, uncorrected baseline emissions inventory.

Uncorrected 2022 baseline emissions inventory before observational correction. Panel **(a)** maps total $PM_{2.5}$-equivalent emissions flux across the contiguous United States, and panels **(b-f)** show precursor-specific sector and release-layer composition as in *Fig. S1.* The overall sectoral structure is broadly similar before and after correction, indicating that the observational adjustment primarily redistributes the magnitude and spatial placement of emissions, especially in high pollution places like California's Central Valley, rather than fundamentally changing the precursor-sector makeup of the inventory.

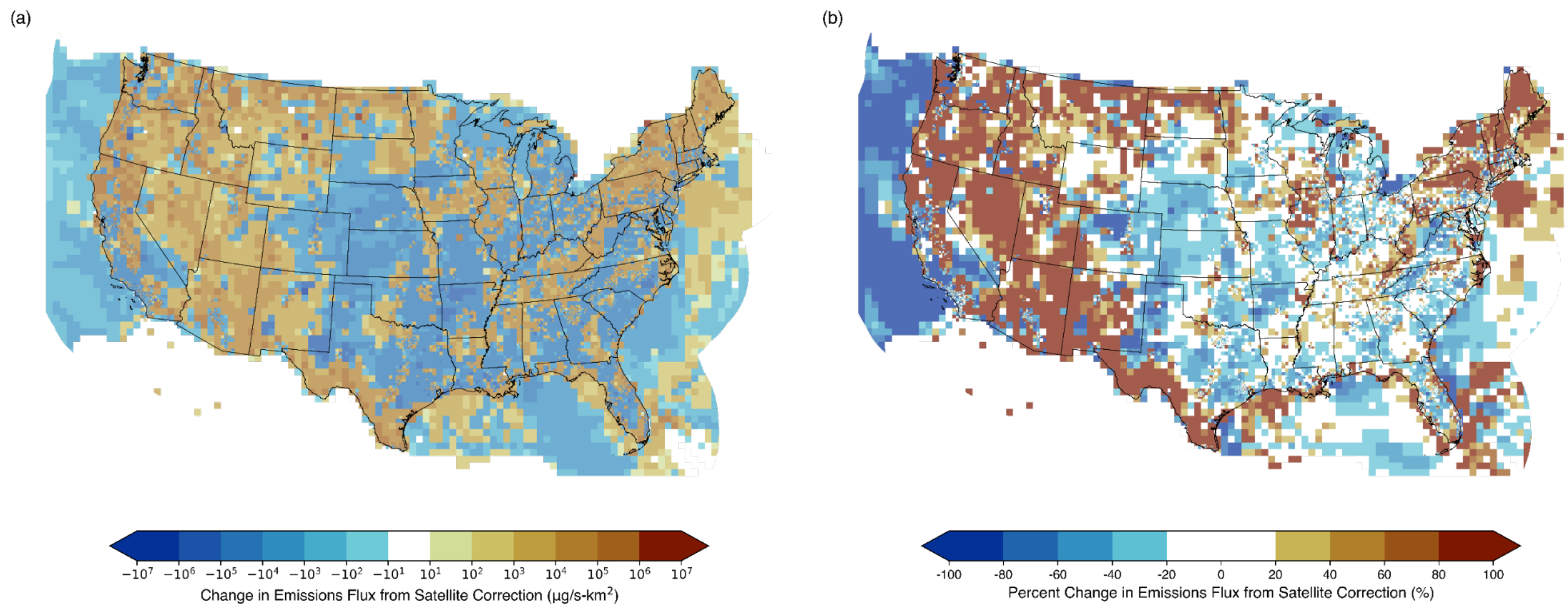

Fig. S5. Differences in emission fluxes after the baseline correction.

Grid-cell (a) absolute and (b) relative differences in $PM_{2.5}$-equivalent emissions flux between the uncorrected National Emissions Inventory baseline and the observationally corrected baseline. Warm colors indicate locations where inferred emissions increased after correction, and cool colors indicate locations where inferred emissions decreased. The correction generally shifts emissions away from some rural locations and toward more densely populated or previously underrepresented hotspots, with notable increases in parts of the Pacific Northwest and New England and decreases across portions of the Midwest.

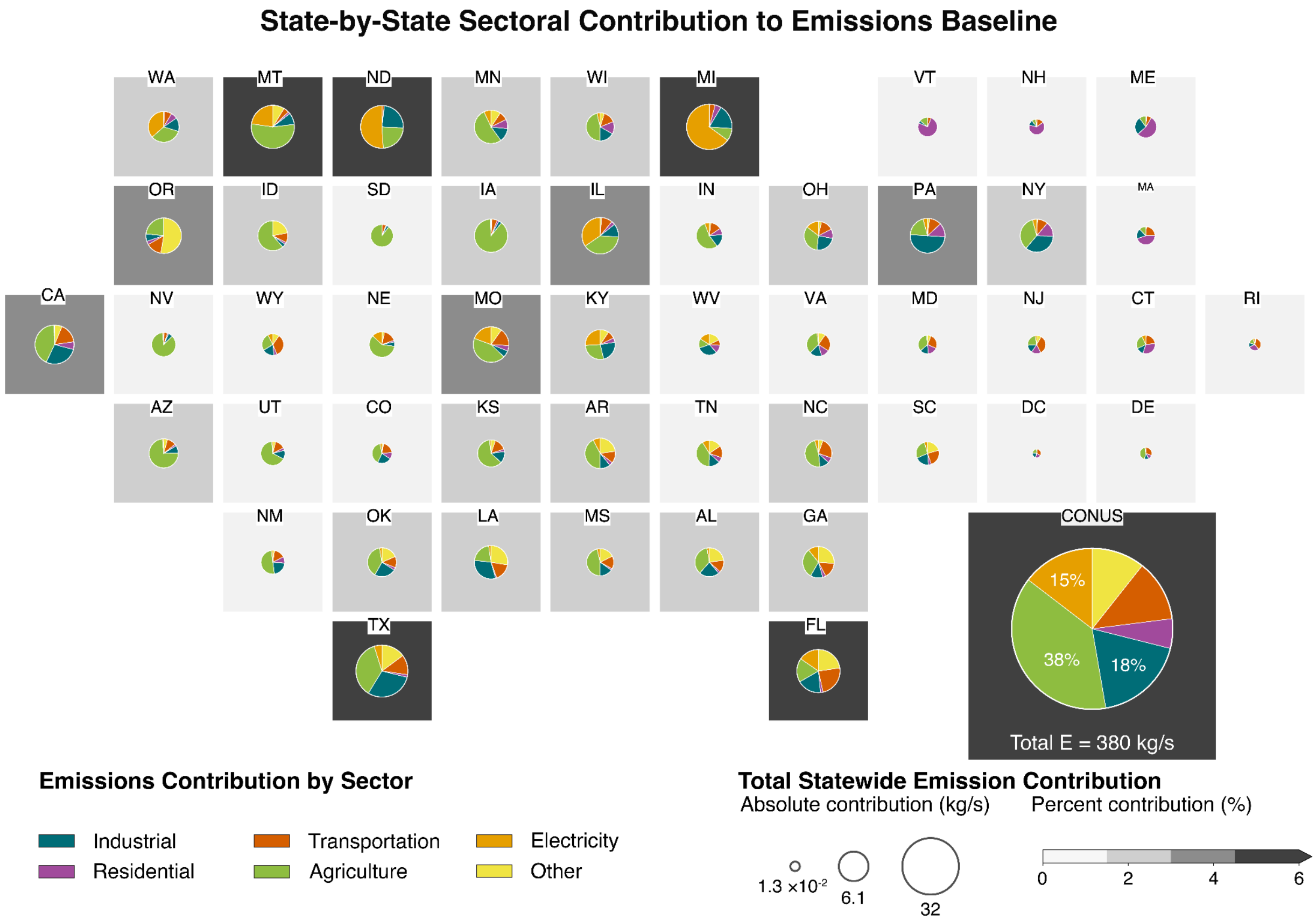

Fig. S6. State-by-state sectoral decomposition of baseline emissions.

State-level baseline emissions shown in the same tiled format as Fig. 4 of the main text. Pie area scales with the absolute magnitude of each state's baseline $PM_{2.5}$-equivalent emissions, while tile shading indicates that state's percentage contribution to the total CONUS baseline inventory. Wedge colors denote sectoral composition. At the national scale, baseline emissions are dominated by Food and Agriculture (38%), followed by Industrial (18%) and Electricity (15%), with smaller but still substantial contributions from Transportation, Other, and Residential sources. The baseline inventory is broadly distributed across the country, but a relatively small number of states account for a disproportionate share of total emissions, including large contributors such as Texas, Florida, Michigan, North Dakota, Montana, and California. The sectoral makeup also varies strongly by state: agricultural emissions dominate many western, plains, and southern states; industrial emissions are more prominent in parts of the Midwest and Northeast; electricity-related emissions make especially large contributions in several central and upper-midwestern states; and Other sources are notably important in states such as Oregon. This figure provides the baseline reference for interpreting the inverse-model reductions in *Fig. 4* and *Figs. S14-S18*, and makes clear that the states that emit the most are not necessarily the same states that the inverse model identifies as carrying the largest attainment burden.

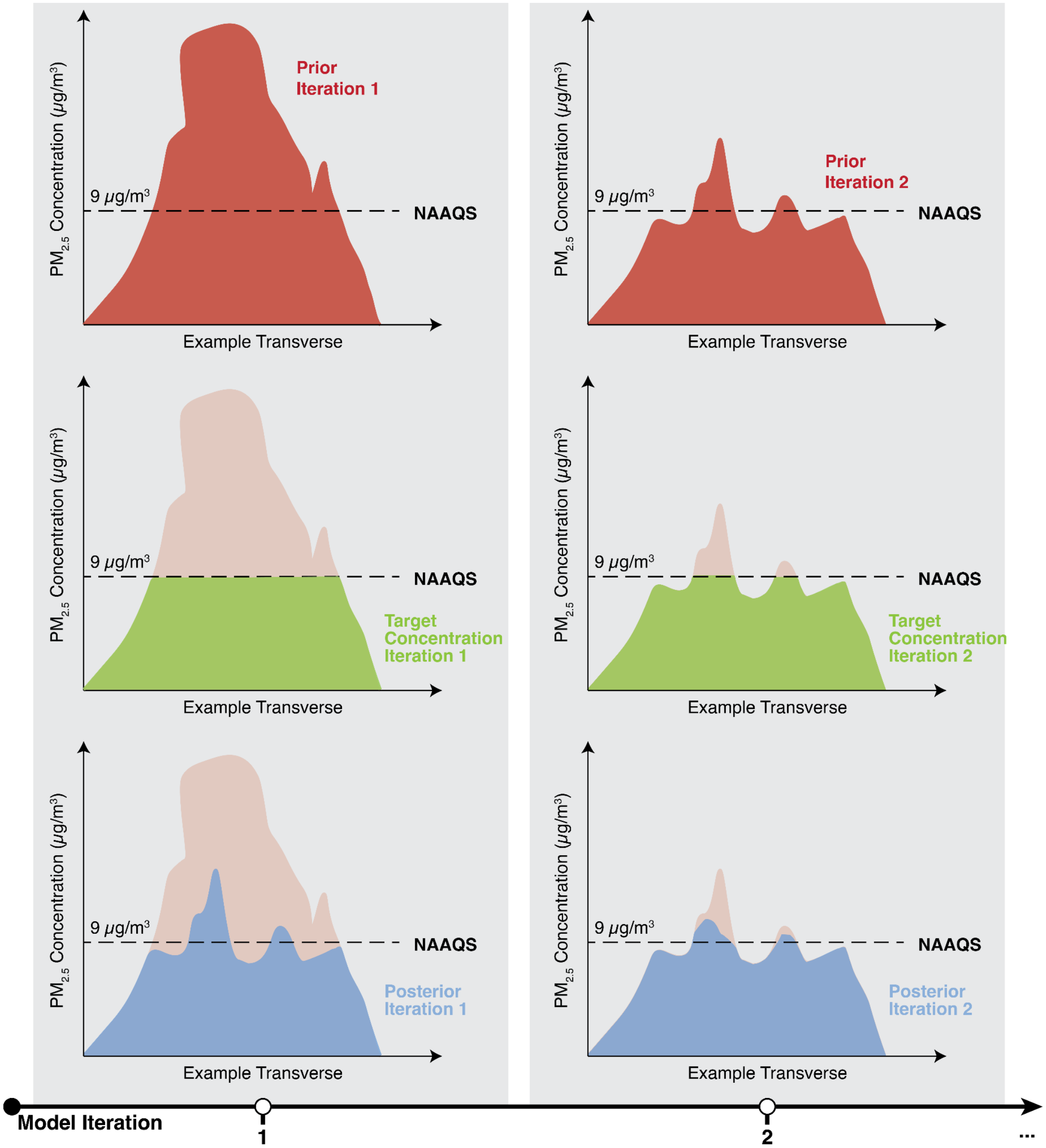

Fig. S7. Schematic illustration of the flexible attainment constraint.

Schematic illustration of the iterative flexible-constraint formulation used to impose attainment without penalizing over-control. Two hypothetical model iterations are shown along an example transect. In each iteration, receptors above the NAAQS are capped at the standard (9 µg/m^3) in the target field, whereas receptors already below the standard are left unconstrained. The updated posterior concentration field from one iteration becomes the prior field for the next iteration. This procedure allows concentrations to fall below the standard where needed to achieve a feasible global solution, while preserving the primary requirement that all exceedances be eliminated at convergence.

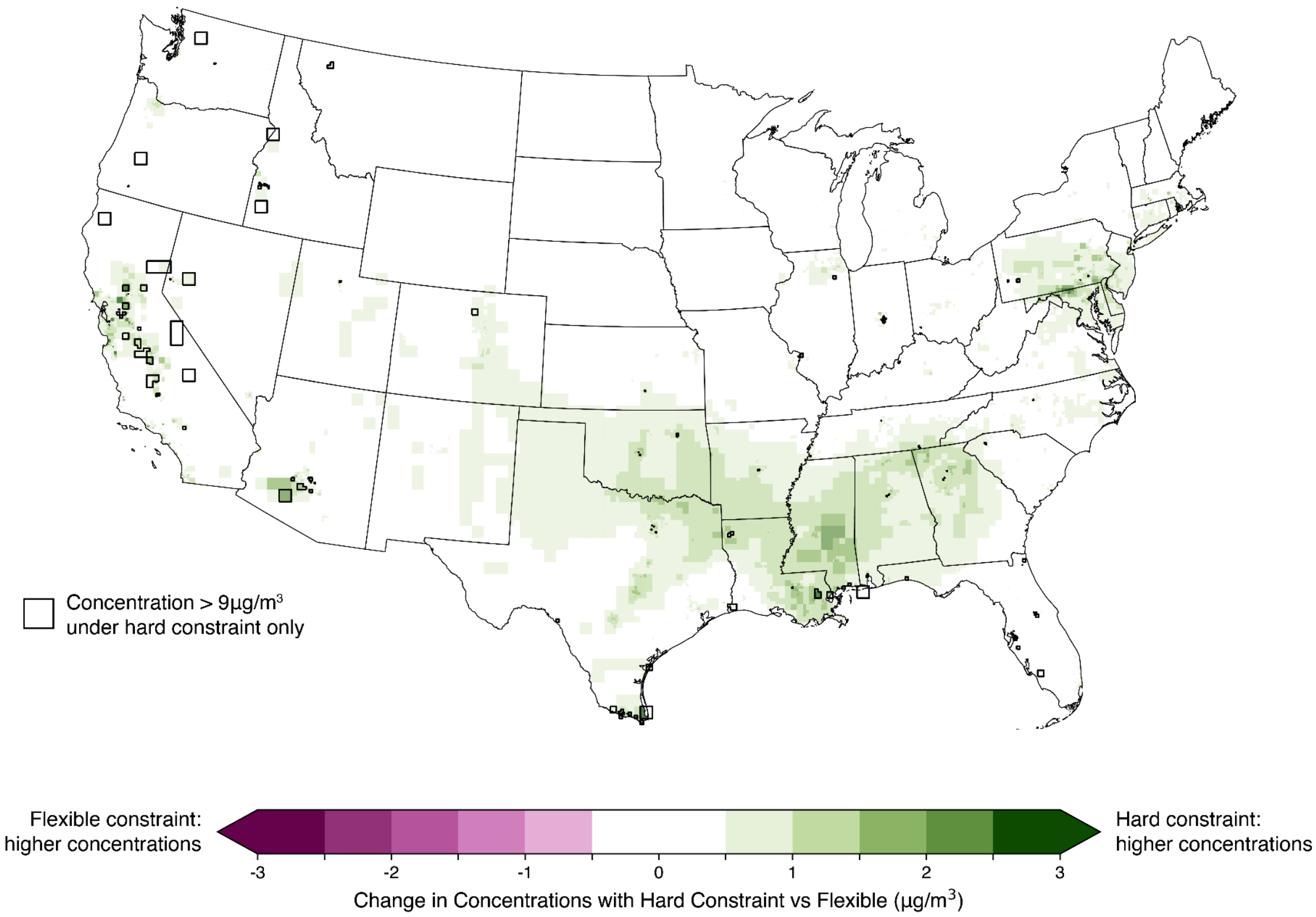

Fig. S8. Flexible attainment targets prevent residual exceedances that persist under a hard equality-style constraint.

Map shows the receptor-wise difference in post-control annual $PM_{2.5}$ concentrations between the hard-constraint and flexible-constraint inversions, plotted as hard minus flexible (µg/m^3). Green shading indicates locations where the hard-constraint formulation yields higher concentrations than the flexible formulation, while magenta indicates the opposite. Open squares denote receptors that remain above 9 µg/m^3 under the hard constraint only. Differences are predominantly positive and are concentrated in major nonattainment regions, including California, the Gulf Coast, parts of the Southeast, and the urbanized Northeast corridor, showing that the hard formulation generally achieves less complete reduction of the upper tail of the concentration field. This pattern arises because the hard equality-style target begins to penalize further reductions once some receptors fall below 9 µg/m^3, even when those same coupled reductions are needed to bring nearby, more strongly violating receptors into compliance. The flexible formulation instead treats 9 µg/m^3 as an upper bound, allowing shared reductions to continue where necessary and thereby eliminating exceedances that would otherwise remain under the hard constraint.

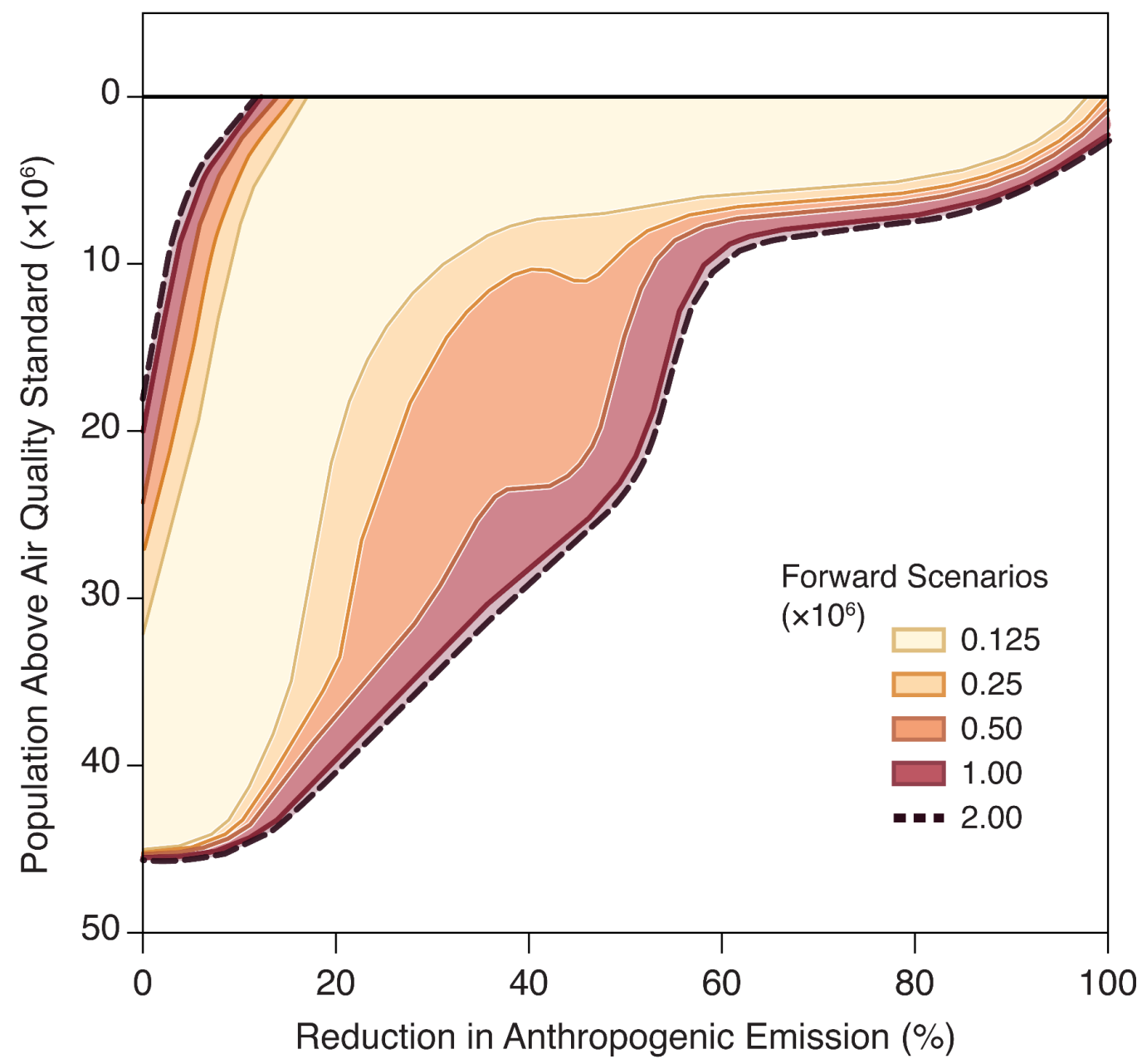

Fig. S9. Effect of the number of forward simulations on the range of identified solutions.

The shaded envelopes show the combinations of anthropogenic emission reductions and population remaining above the annual $PM_{2.5}$ standard found using 0.125, 0.25, 0.50, and 1.00 million simulations; the dashed line shows the 2-million-simulation boundary. The x-axis shows the percent reduction in anthropogenic emissions, and the inverted y-axis shows the population above the standard (i.e., better air-quality outcomes appear higher). Smaller ensembles miss substantial parts of the solution space, especially at intermediate emission reductions. The sampled space expands considerably up to 0.5 million runs, but the additional coverage becomes progressively smaller. The 1-million-run envelope is close to the 2-million-run boundary across most of the figure, showing that one million simulations capture the main range and shape of possible outcomes.

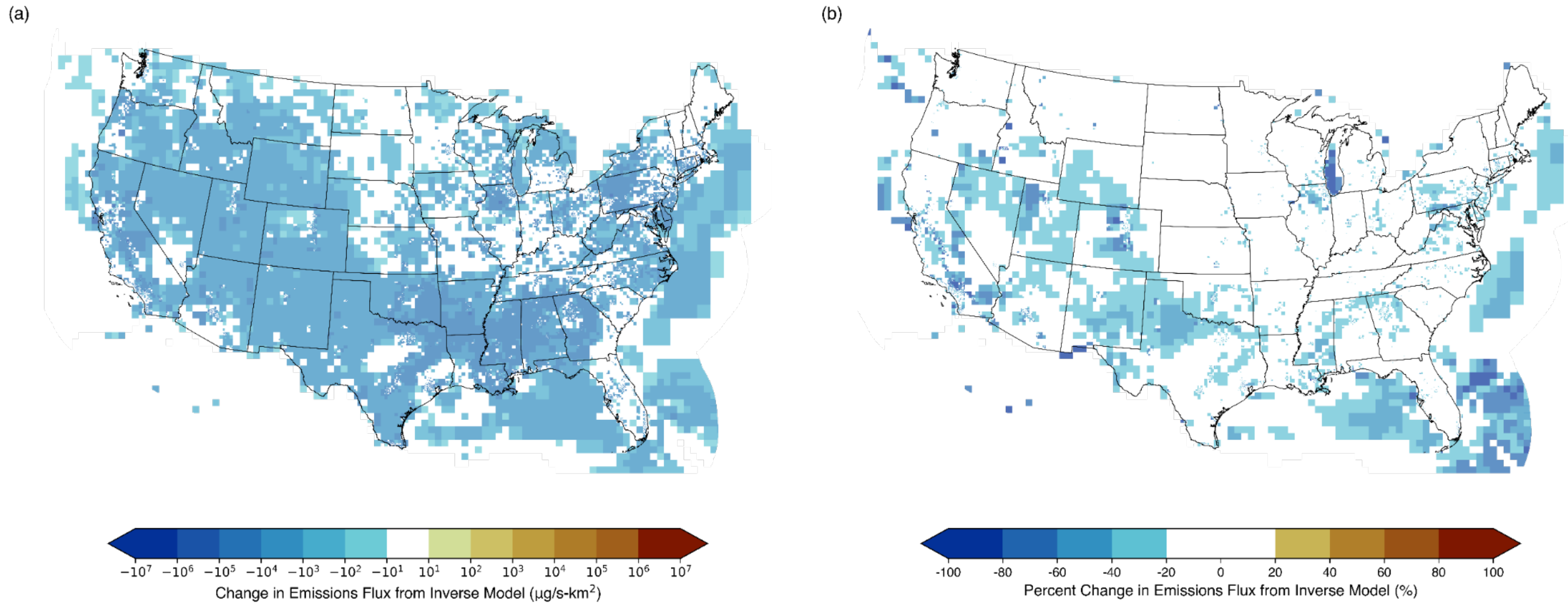

Fig. S10. Differences in grid-level emission fluxes from the corrected baseline to the inverse model solution.

Grid-cell (a) absolute and (b) relative changes in $PM_{2.5}$-equivalent emissions flux between the corrected baseline inventory and the inverse-model attainment solution. Because the inverse problem is constrained to monotonic controls, no grid cell shows an emissions increase. The resulting pattern is characterized by widespread but generally modest reductions across much of the domain, with relatively few grid cells requiring very large reductions. This spatial structure reflects the fact that minimum-intervention attainment is achieved through many distributed, high-leverage adjustments rather than through extreme control in only a small number of places.

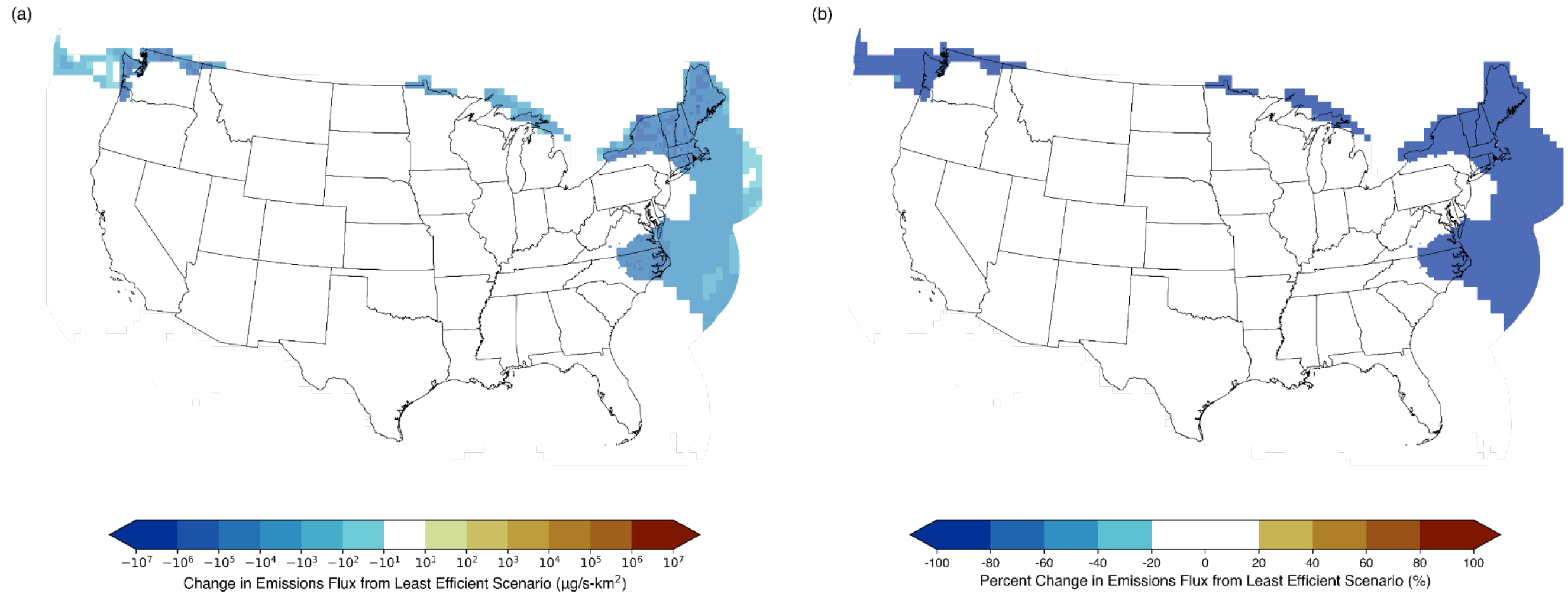

Fig. S11. Differences in emission fluxes from the corrected baseline to the Least Efficient scenario model solution.

Grid-cell (a) absolute and (b) relative changes in $PM_{2.5}$-equivalent emissions flux between the corrected baseline inventory and the Least Efficient Scenario highlighted in Fig. 3. This scenario applies the same aggregate reduction in controllable emissions as the inverse solution but allocates those reductions to locations with minimal leverage for attainment. Most of the emission reductions are located in the northernmost rural parts of the Continental US and in the Atlantic Ocean downwind of coastal communities. While the emission reductions in parts of New England, Virginia, and North Carolina likely reduce population exposures, they do very little to reduce NAAQS exceedances.

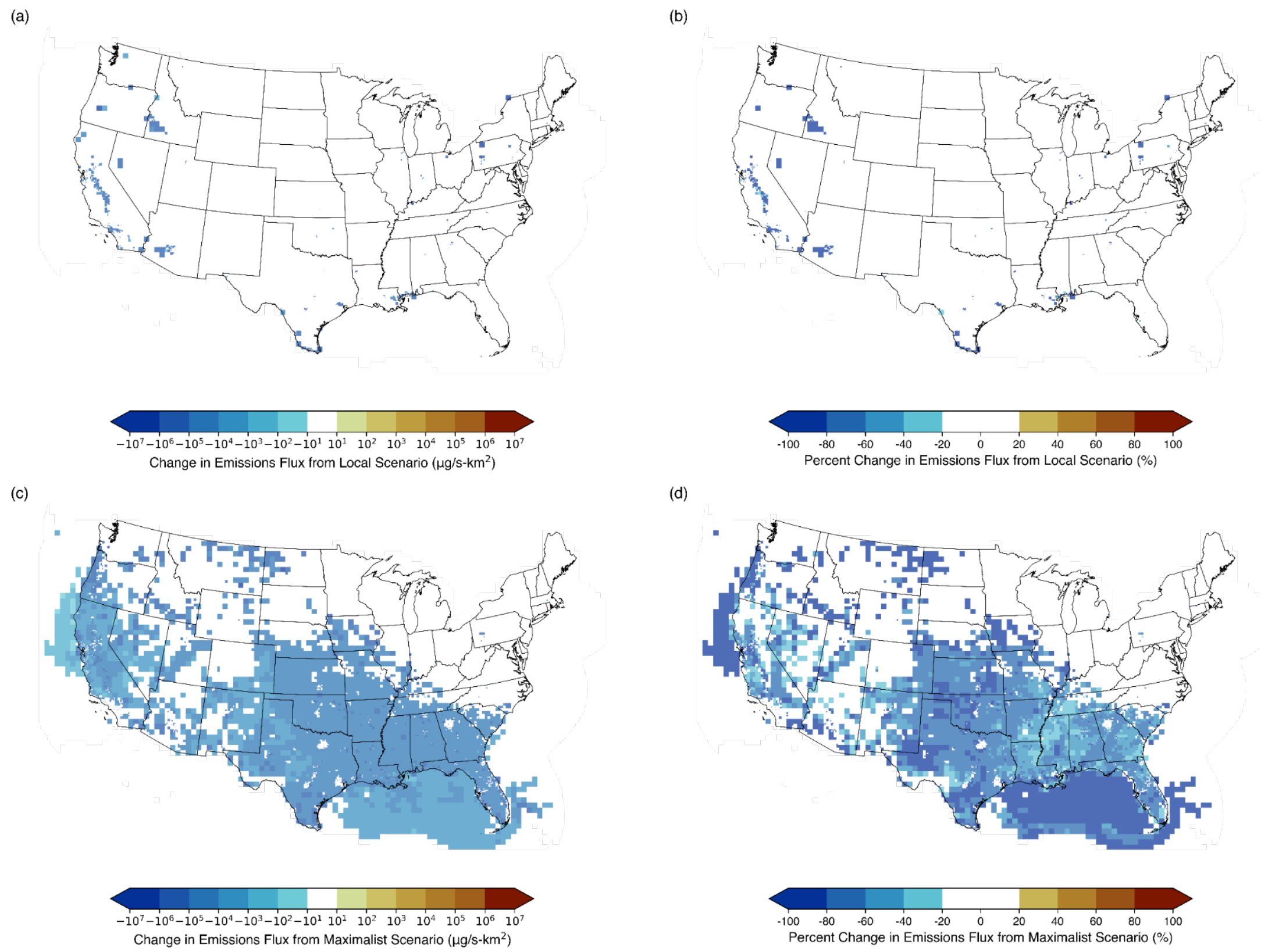

Fig. S12. Differences in emission fluxes from the corrected baseline to the Local and Maximalist attainment scenarios.

Absolute and relative grid-cell changes in $PM_{2.5}$-equivalent emissions flux between the corrected baseline inventory and two simplistic attainment algorithms: **(a-b)** the "Local Solution" and **(c-d)** the "Maximalist Solution". Both maps are shown relative to the same corrected baseline. The "Local Solution" is highly localized because it simply zeros controllable emissions in baseline nonattainment grid cells, without accounting for the broader source-receptor structure. This produces concentrated reductions in the initially violating locations while leaving most of the domain unchanged. By contrast, the "Maximalist Solution" produces broad, spatially diffuse reductions across much of the western, south-central, and southeastern US, reflecting its sequential attainment-oriented logic: it repeatedly selects the next most effective control for reducing remaining exceedances, but ultimately requires progressively wider system-wide reductions to eliminate hard-to-reach receptors. Together, these panels illustrate two distinct failure modes of heuristic control design: the "Local Solution" under-represents the spatially distributed source contributions that govern attainment, whereas the "Maximalist Solution" over-controls broad regions of the system relative to the globally efficient inverse-derived allocation.

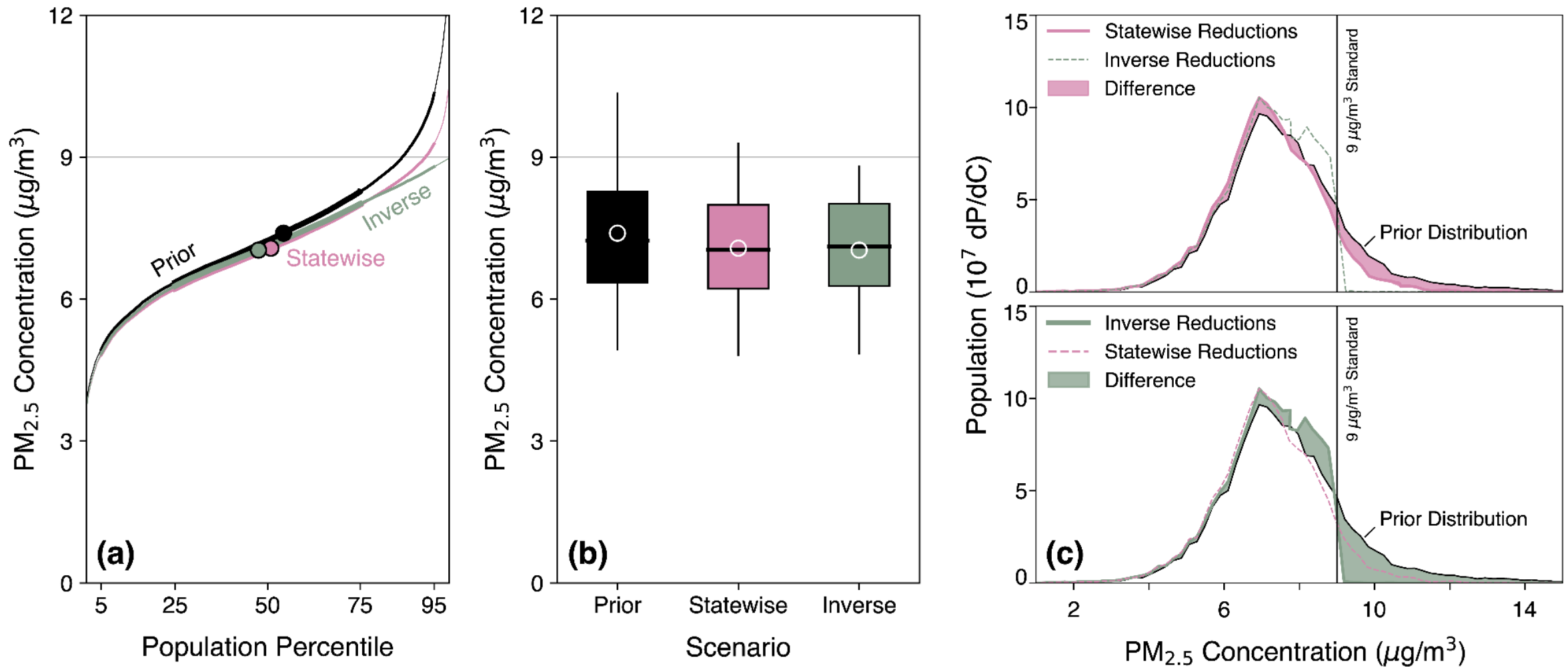

Fig. S13. Comparison of the grid-resolved inverse solution and a statewise reallocated solution.

Comparison of the fully grid-resolved inverse solution with a statewise reallocated scenario that preserves the same total sector-specific reductions in each state but redistributes those reductions uniformly within states. This comparison isolates the value of within-state spatial allocation in the inverse solution. Panel **(a)** shows population-percentile concentration curves for the prior, the statewise reallocated solution, and the original inverse solution. The two control scenarios closely track one another over much of the distribution, but they diverge in the upper tail, where the grid-resolved inverse solution more effectively suppresses the highest concentrations. Panel **(b)** summarizes the resulting concentration distributions across grid cells, showing that the statewise and inverse solutions have broadly similar central tendencies despite meaningful differences in the most attainment-relevant portion of the distribution. Panel **(c)** compares the corresponding population exposure distributions relative to the prior state. Both control scenarios shift the prior distribution leftward, but the inverse solution moves more population from just above the 9 μg/m^3 standard to just below it, whereas the statewise reallocated solution leaves a somewhat larger near-threshold and above-threshold tail. Taken together, these panels show that resolving controls at the grid-cell level yields the most efficient attainment pathway, but they also show that much of the structure identified by the inverse model is already captured at the level of states and sectors. In that sense, the grid-resolved inverse solution should be interpreted as a reference benchmark for minimum-intervention attainment, while the statewise decomposition can provide a more interpretable starting point for policy discussion and subsequent forward evaluation.

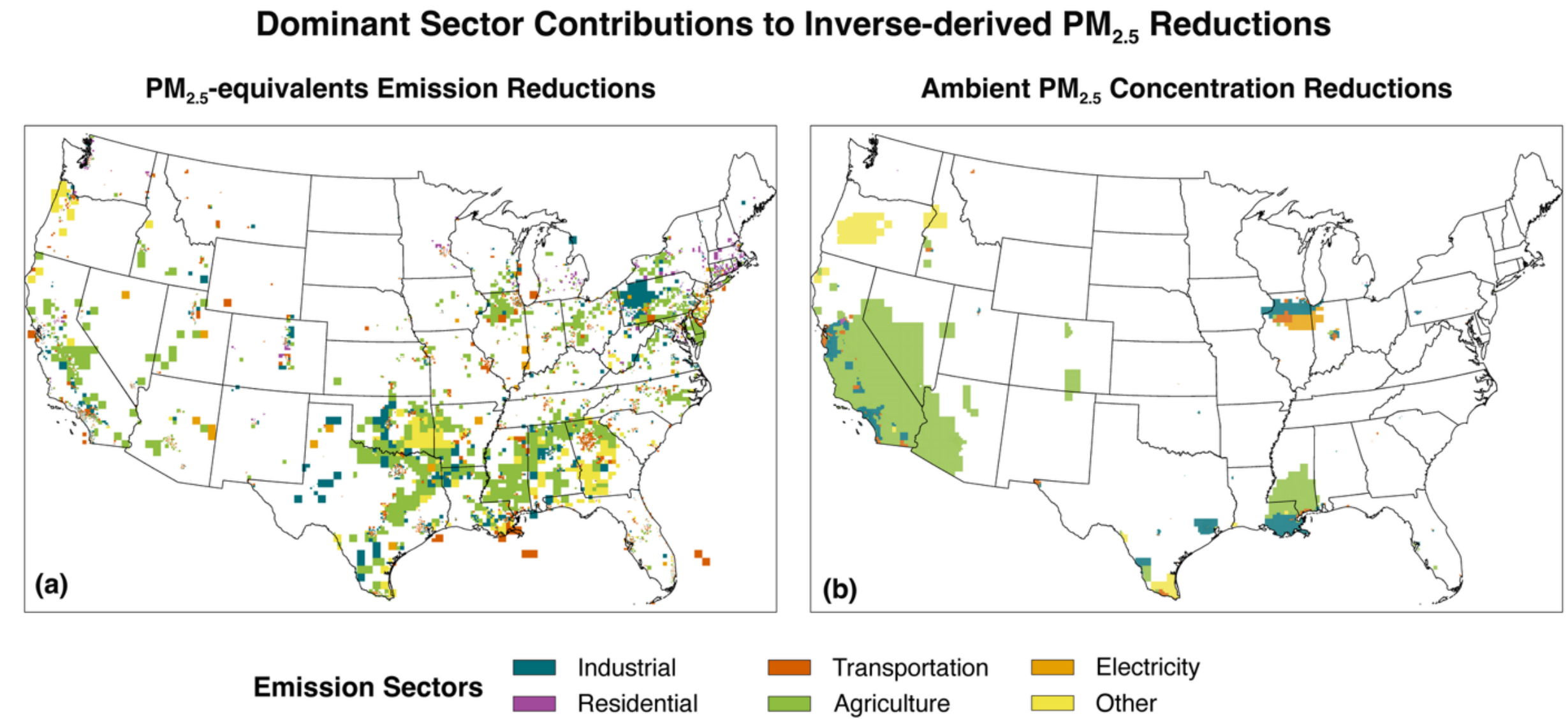

Fig. S14. Sectors contributing most strongly to inverse-derived emission and $PM_{2.5}$ concentration reductions.

Colors identify the sector making the largest contribution in each displayed grid cell. **(a)** Agriculture drives emission reductions across broad areas of the West, central United States, and Southeast, although industrial, transportation, electricity, and other sources are important in many locations. **(b)** The largest $PM_{2.5}$ concentration reductions are more geographically concentrated and show a different sectoral pattern. Agriculture remains important across much of California, while industrial sources dominate several areas along the Gulf Coast, in California, and around the Midwest. The contrast shows that the sector with the largest emission reduction does not always provide the largest local air-quality improvement because emissions from different locations and sectors affect downwind $PM_{2.5}$ differently.

Figs. S15-S19 uses the same state-level visualization as **Fig. 4** in the main text, but disaggregate the cumulative $PM_{2.5}$-equivalent attainment burden into precursor-specific reductions. Across all five figures, pie area scales with the absolute emissions reduction required in each state for that precursor, tile shading indicates the percent reduction relative to the state's baseline emissions for that precursor, and wedge colors denote sectoral contributions. Whereas **Fig. 4** summarizes the total inverse-model solution in $PM_{2.5}$-equivalent units, **Figs. S15-S19** show which precursor controls underlie that aggregate pattern.

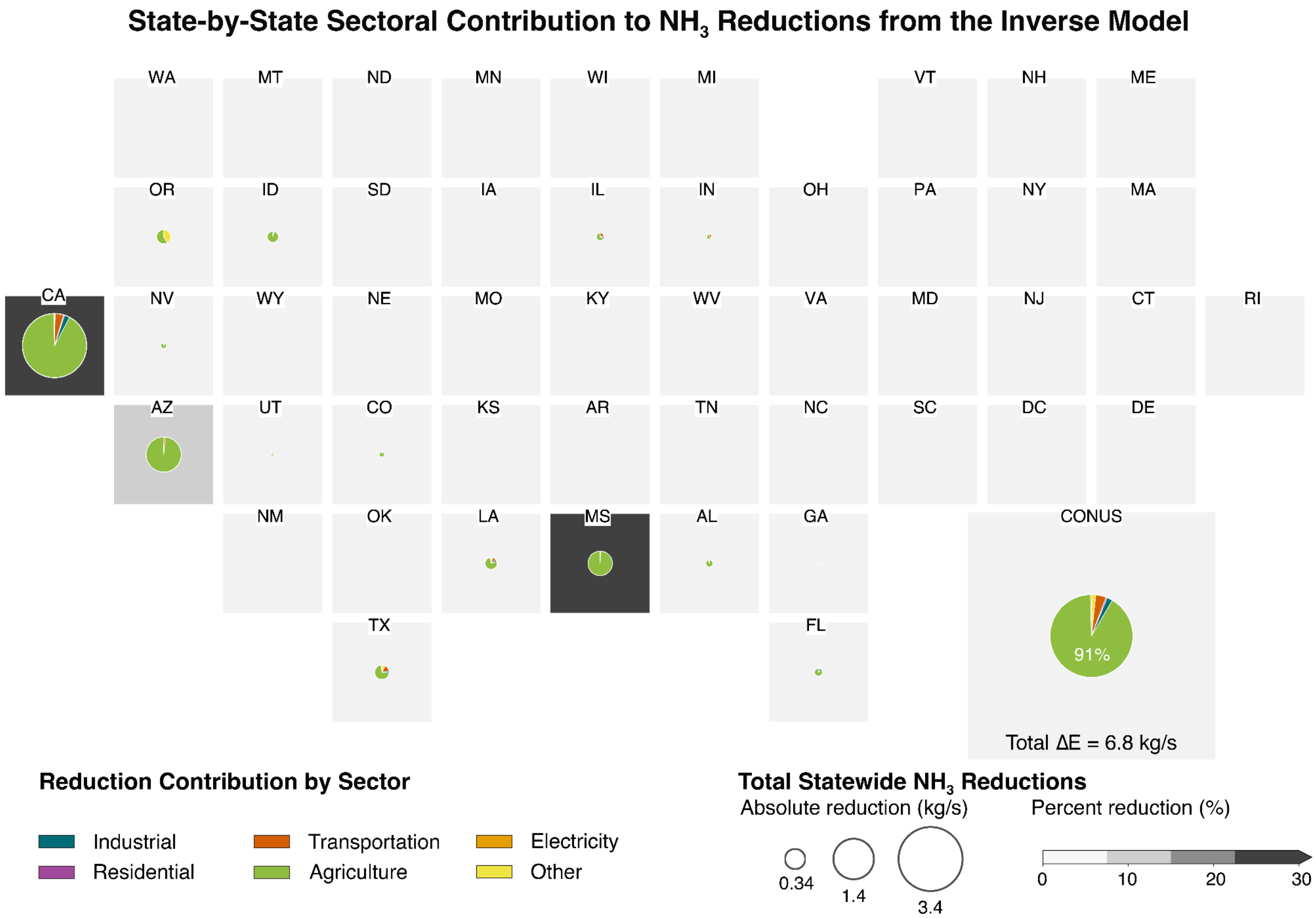

Fig. S15. State-by-state sectoral contribution to NH_3 reductions from the inverse model.

Relative to the mixed-sector pattern in main-text **Fig. 4**, the NH_3-resolved solution is much more sectorally concentrated: reductions are overwhelmingly dominated by the Food and Agriculture sector nationwide. The largest absolute NH_3reductions occur in California and Arizona, with smaller contributions from a limited set of additional agricultural states, showing that the NH_3 component of the cumulative attainment burden is both geographically concentrated and sectorally narrow.

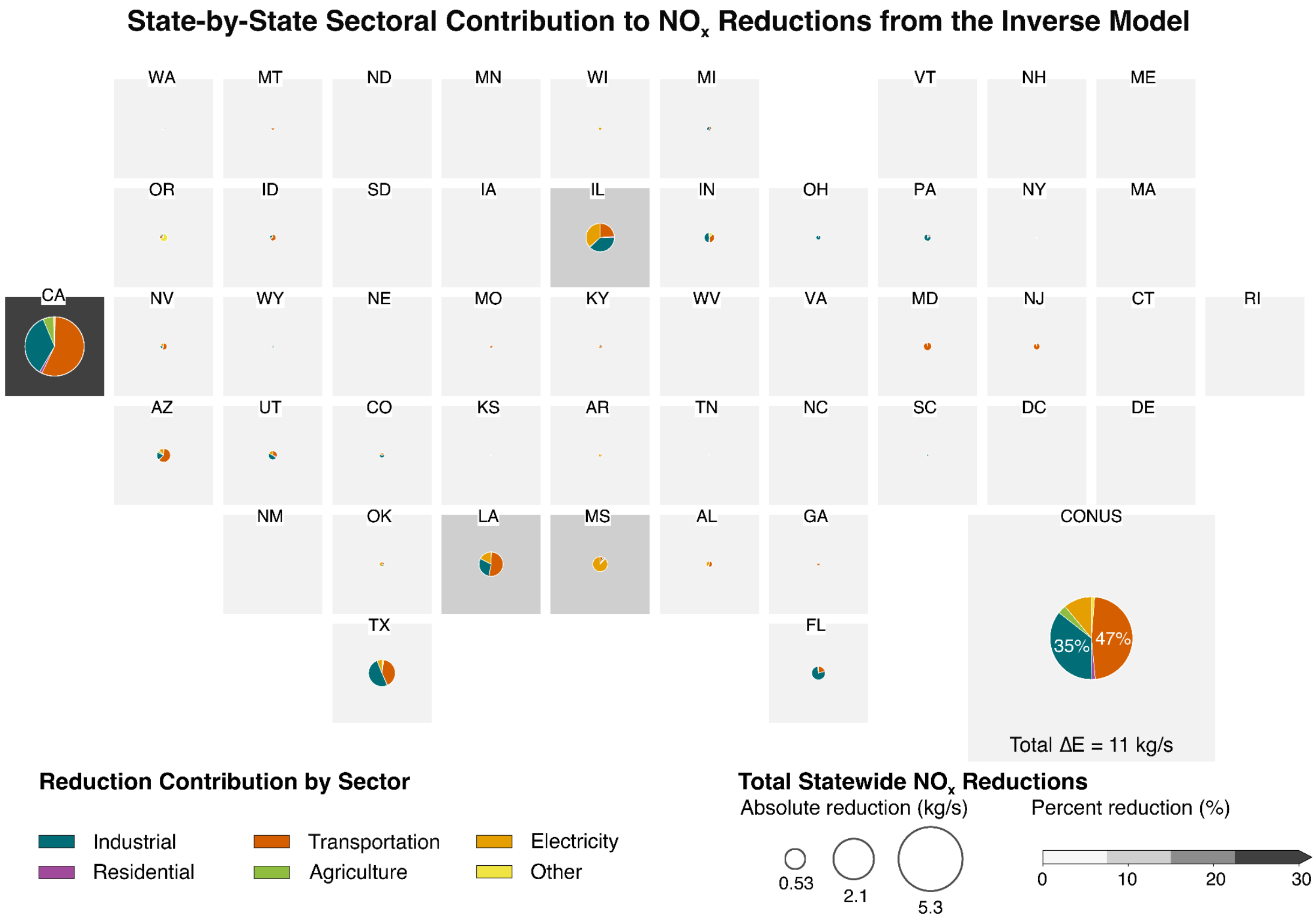

Fig. S16. State-by-state sectoral contribution to NO_x reductions from the inverse model.

In contrast to the aggregate $PM_{2.5}$-equivalent view in **Fig. 4**, the NO_x-resolved pattern reveals a broader and more spatially distributed control burden. NO_x reductions are shared primarily between Transportation and Industrial sources, with additional Electricity contributions in selected states. Large reductions in California, Texas, Illinois, Louisiana, and Florida show that part of the cumulative burden in **Fig. 4** reflects widespread NO_x controls tied to urban transport corridors and industrial activity rather than a single dominant source class.

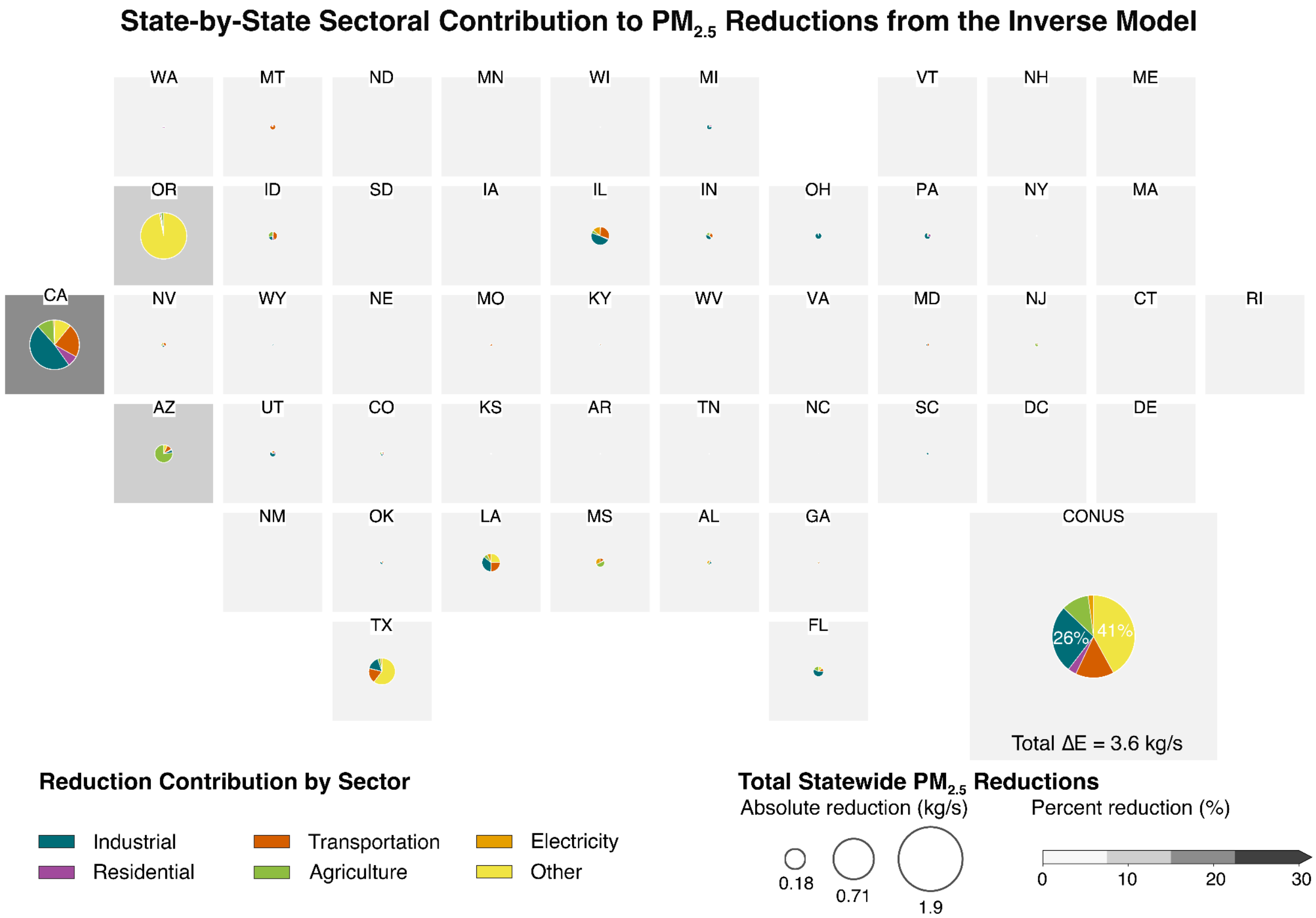

Fig. S17. State-by-state sectoral contribution to primary $PM_{2.5}$ reductions from the inverse model.

Compared with the cumulative $PM_{2.5}$-equivalent solution in **Fig. 4**, the direct primary $PM_{2.5}$ component is smaller in total magnitude and more uneven in composition. The national reduction is driven strongly by the Other sector, with additional Industrial and Transportation contributions. Oregon stands out for a particularly large relative contribution from Other sources, while California and Texas remain important in absolute terms, indicating that direct particulate controls account for a more selective subset of the broader attainment burden summarized in **Fig. 4**.

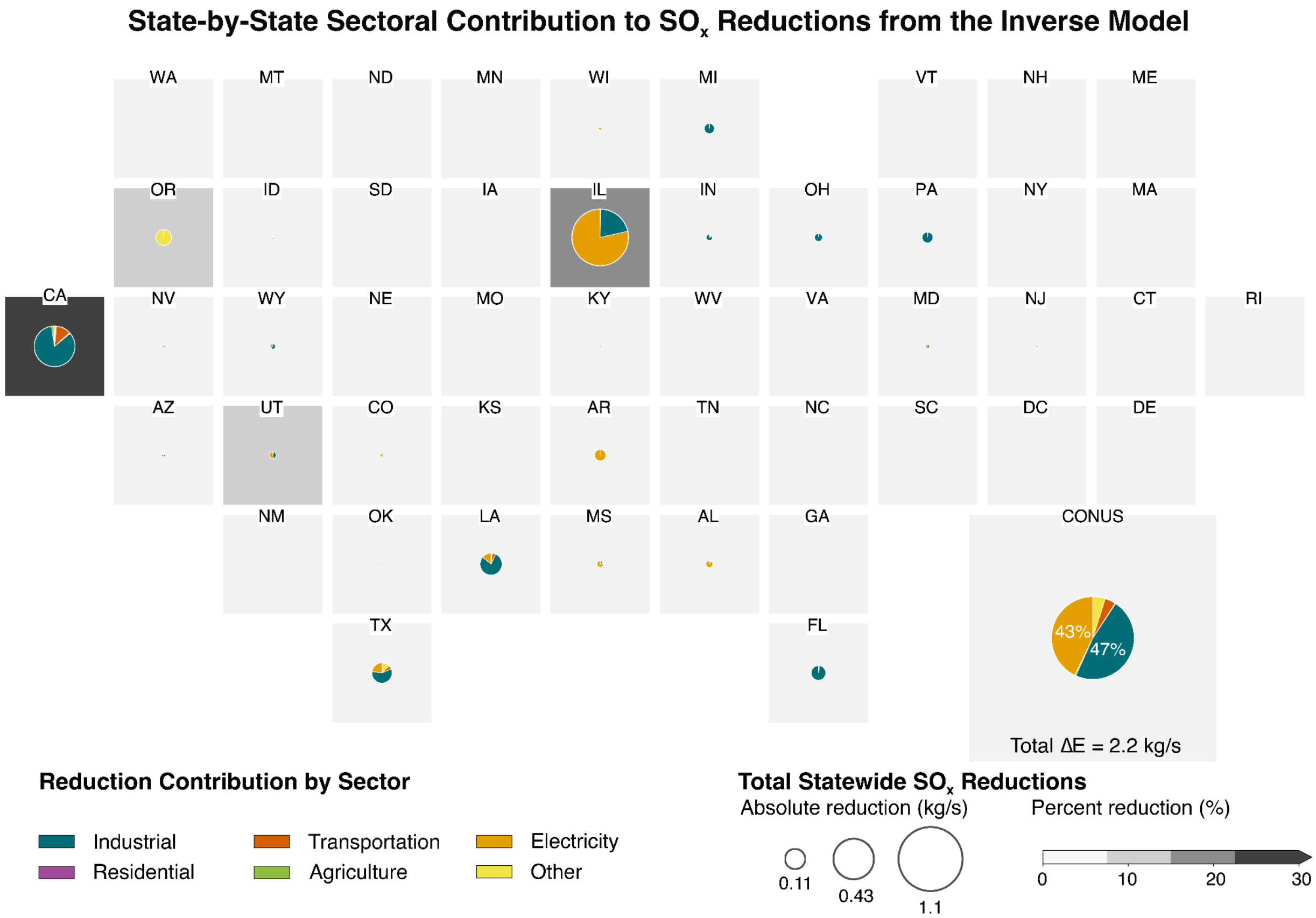

Fig. S18. State-by-state sectoral contribution to SO_x reductions from the inverse model.

Where **Fig. 4** shows a broad cumulative attainment burden across several sectors, the SO_x-resolved view isolates one of its most spatially concentrated components. SO_x reductions are dominated by Industrial and Electricity-sector controls, with Illinois standing out as the clearest hotspot in both absolute and relative reduction burden and additional contributions from California, Louisiana, Texas, and Florida. This indicates that the sulfur-related portion of the aggregate inverse solution is localized in a relatively small number of power- and industry-intensive states.

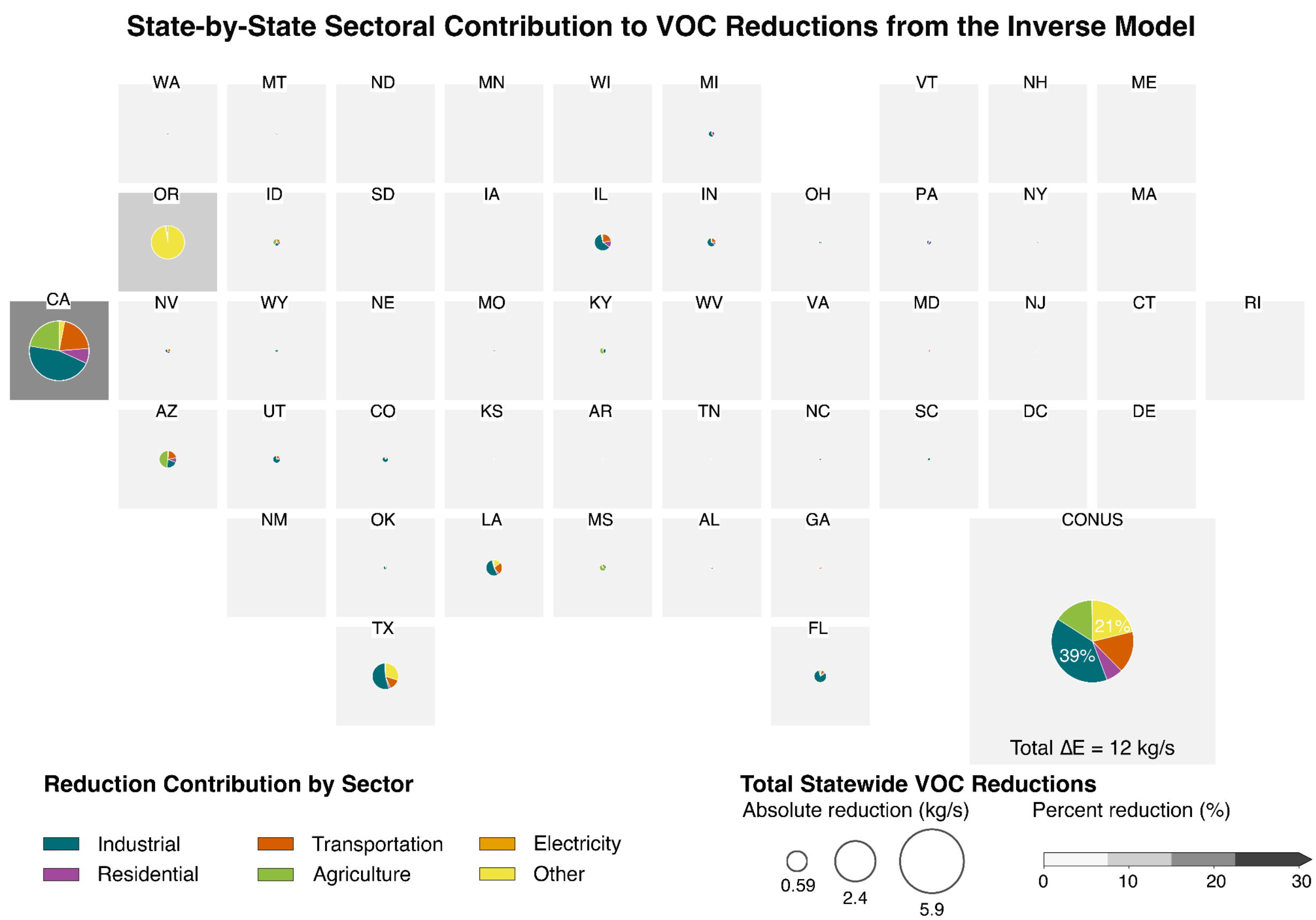

Fig. S19. State-by-state sectoral contribution to VOC reductions from the inverse model.

VOC reductions represent one of the largest precursor-specific components underlying the cumulative $PM_{2.5}$-equivalent burden shown in **Fig. 4**. Unlike NH_3 or SO_x, the VOC pattern is both geographically broad and sectorally mixed, with especially large Industrial contributions and meaningful roles for Other, Transportation, Residential, and Agriculture in different states. California and Texas account for the largest absolute VOC reductions, while Oregon, Louisiana, Illinois, and Florida also contribute noticeably, showing that a substantial fraction of the aggregate control burden in **Fig. 4** is carried through diverse VOC-related pathways.

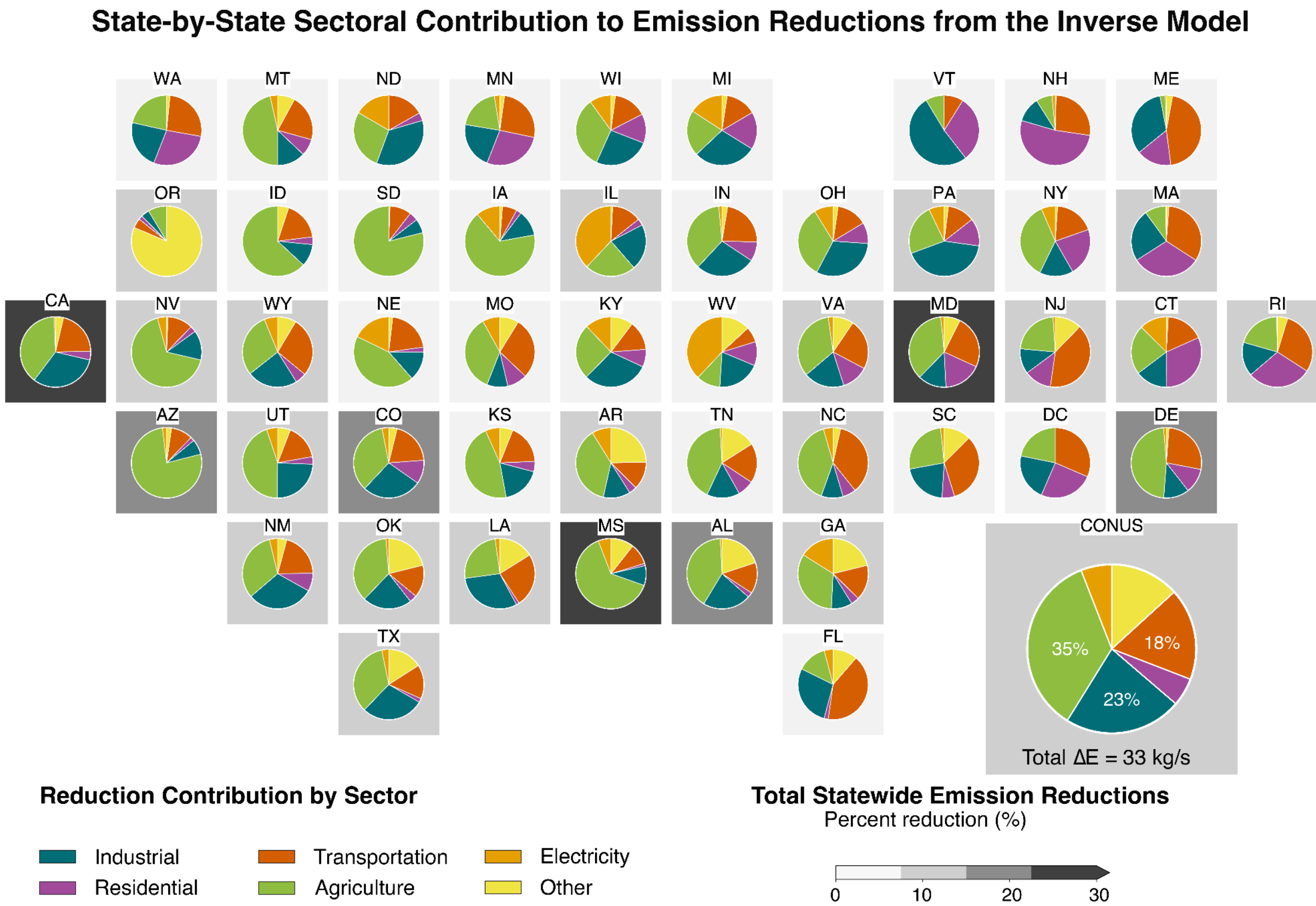

Fig. S20. State-by-state sectoral decomposition of emissions reductions required by the inverse solution, shown with uniformly sized pie charts for visual comparability.

Each main tile represents a single state's inverse model results aggregated to the state level. In contrast to **Fig. 4**, all pie charts are shown at the same size to improve visibility of sectoral composition across states, independent of the absolute magnitude of emissions reduction. Tile shading indicates the percent emissions reduction relative to that state's baseline emissions. Wedge colors denote the sectoral contribution to each state's required reduction: industrial, residential, transportation, agriculture, electricity, and other. The CONUS summary pie is shown at the bottom right.

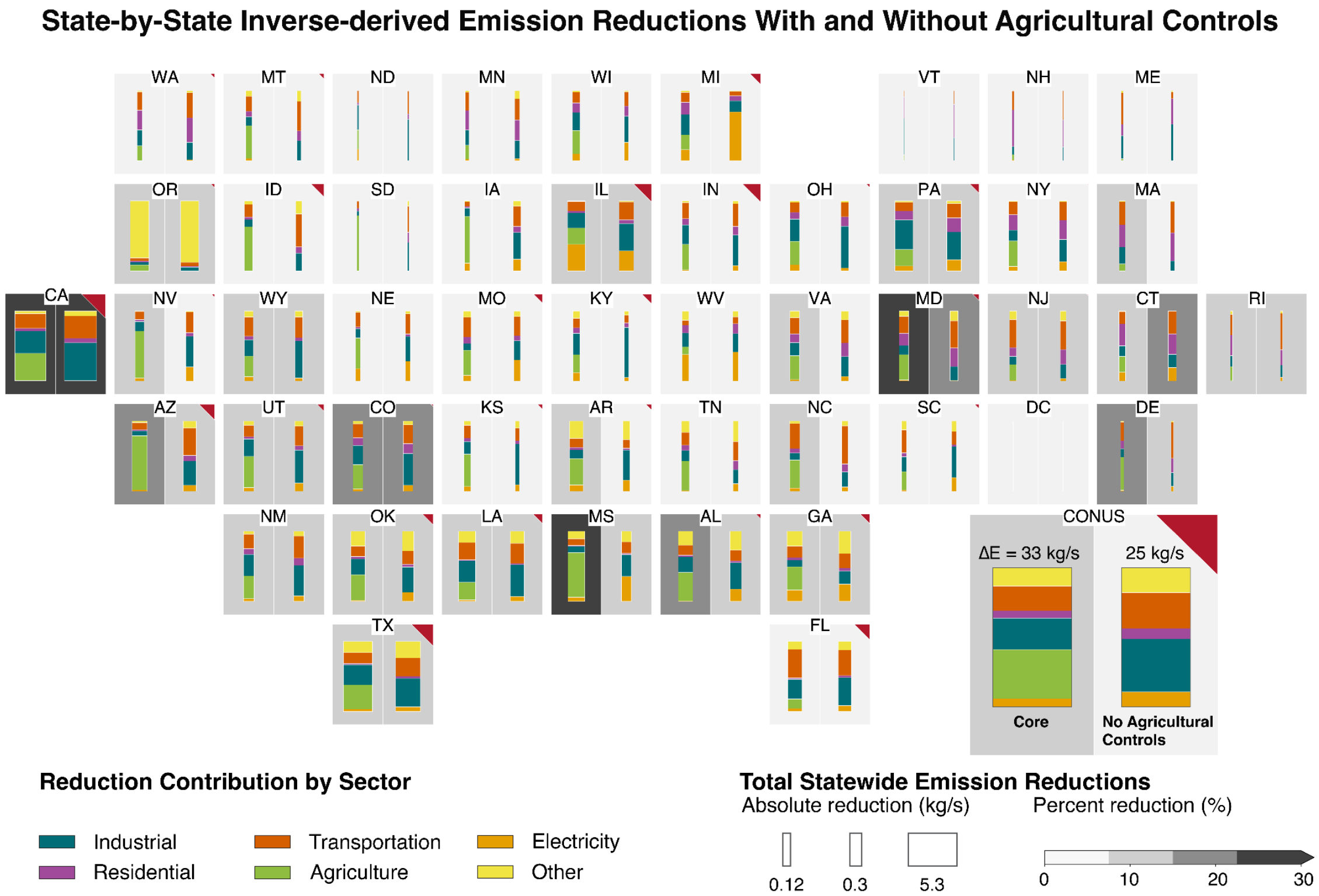

Fig. S21. State-level magnitude and sectoral composition of optimized emission reductions under the core and no-agricultural-control cases.

Each state contains paired stacked bars for the core optimization (left) and the case in which agricultural emissions are not controlled (right). Bar width represents the absolute state-level $PM_{2.5}$-equivalent emission reduction, while the colored segments show the relative contributions of each sector. Background shading indicates the percentage reduction from the state's anthropogenic emissions baseline. Red corner markers identify population remaining above the annual $PM_{2.5}$ NAAQS of 9 μg/m^3, with marker area proportional to the affected population. Removing agricultural controls reduces the optimized CONUS-wide emission reduction from 33 to 25 kg/s and shifts reductions toward industrial, transportation, and other sources. More importantly, the population remaining above the NAAQS increases from zero in the core case to 6.1 million people. In these locations, the minimum concentration attainable through controls on the remaining sectors is already above the NAAQS, meaning attainment cannot be achieved without agricultural emission reductions.

SI References

(1) Tessum, C. W.; Hill, J. D.; Marshall, J. D. InMAP: A Model for Air Pollution Interventions. *PLoS ONE* **2017**, *12* (4), e0176131. https://doi.org/10.1371/journal.pone.0176131.

(2) Goodkind, A. L.; Tessum, C. W.; Coggins, J. S.; Hill, J. D.; Marshall, J. D. Fine-Scale Damage Estimates of Particulate Matter Air Pollution Reveal Opportunities for Location-Specific Mitigation of Emissions. *Proc Natl Acad Sci USA* **2019**, *116* (18), 8775–8780. https://doi.org/10.1073/pnas.1816102116.

(3) Goodkind, A. L.; Tessum, C. W.; Coggins, J. S.; Hill, J. D.; Marshall, J. D. InMAP Source-Receptor Matrix (ISRM) Dataset, 2019. https://doi.org/10.5281/ZENODO.2589759.

(4) US Environmental Protection Agency. National Emissions Inventory 2017. https://www.epa.gov/air-emissions-inventories/national-emissions-inventory-nei (accessed 2025-06-27).

(5) McDuffie, E. E.; Martin, R. V.; Spadaro, J. V.; Burnett, R.; Smith, S. J.; O'Rourke, P.; Hammer, M. S.; van Donkelaar, A.; Bindle, L.; Shah, V.; Jaeglé, L.; Luo, G.; Yu, F.; Adeniran, J. A.; Lin, J.; Brauer, M. Source Sector and Fuel Contributions to Ambient PM2.5 and Attributable Mortality across Multiple Spatial Scales. *Nature Communications* **2021**, *12* (1), 3594. https://doi.org/10.1038/s41467-021-23853-y.

(6) Van Donkelaar, A.; Hammer, M. S.; Bindle, L.; Brauer, M.; Brook, J. R.; Garay, M. J.; Hsu, N. C.; Kalashnikova, O. V.; Kahn, R. A.; Lee, C.; Levy, R. C.; Lyapustin, A.; Sayer, A. M.; Martin, R. V. Monthly Global Estimates of Fine Particulate Matter and Their Uncertainty. *Environ. Sci. Technol.* **2021**, *55* (22), 15287–15300. https://doi.org/10.1021/acs.est.1c05309.

(7) Tessum, C. W.; Hill, J. D.; Marshall, J. D. Twelve-Month, 12 Km Resolution North American WRF-Chem v3.4 Air Quality Simulation: Performance Evaluation. *Geosci. Model Dev.* **2015**, *8* (4), 957–973. https://doi.org/10.5194/gmd-8-957-2015.

(8) Heo, J.; Adams, P. J.; Gao, H. O. Reduced-Form Modeling of Public Health Impacts of Inorganic PM 2.5 and Precursor Emissions. *Atmospheric Environment* **2016**, *137*, 80–89. https://doi.org/10.1016/j.atmosenv.2016.04.026.

(9) Gilmore, E. A.; Heo, J.; Muller, N. Z.; Tessum, C. W.; Hill, J. D.; Marshall, J. D.; Adams, P. J. An Inter-Comparison of the Social Costs of Air Quality from Reduced-Complexity Models. *Environmental Research Letters* **2019**, *14* (7), 074016. https://doi.org/10.1088/1748-9326/ab1ab5.

(10) Rodgers, C. D. *Inverse Methods for Atmospheric Sounding: Theory and Practice*; Series on Atmospheric, Oceanic and Planetary Physics; WORLD SCIENTIFIC, 2000; Vol. 2. https://doi.org/10.1142/3171.

(11) Streets, D. G.; Canty, T.; Carmichael, G. R.; De Foy, B.; Dickerson, R. R.; Duncan, B. N.; Edwards, D. P.; Haynes, J. A.; Henze, D. K.; Houyoux, M. R.; Jacob, D. J.; Krotkov, N. A.; Lamsal, L. N.; Liu, Y.; Lu, Z.; Martin, R. V.; Pfister, G. G.; Pinder, R. W.; Salawitch, R. J.; Wecht, K. J. Emissions Estimation from Satellite Retrievals: A Review of Current Capability. *Atmospheric Environment* **2013**, *77*, 1011–1042. https://doi.org/10.1016/j.atmosenv.2013.05.051.

(12) Yadav, V.; Michalak, A. M. Improving Computational Efficiency in Large Linear Inverse Problems: An Example from Carbon Dioxide Flux Estimation. *Geosci. Model Dev.* **2013**, *6* (3), 583–590. https://doi.org/10.5194/gmd-6-583-2013.

(13) Bergamaschi, P.; Segers, A.; Brunner, D.; Haussaire, J.-M.; Henne, S.; Ramonet, M.; Arnold, T.; Biermann, T.; Chen, H.; Conil, S.; Delmotte, M.; Forster, G.; Frumau, A.; Kubistin, D.; Lan, X.; Leuenberger, M.; Lindauer, M.; Lopez, M.; Manca, G.; Müller-Williams, J.; O'Doherty, S.; Scheeren, B.; Steinbacher, M.; Trisolino, P.; Vítková, G.; Yver Kwok, C. High-Resolution Inverse Modelling of European CH_4 Emissions Using the Novel FLEXPART-COSMO TM5 4DVAR Inverse Modelling System. *Atmos. Chem. Phys.* **2022**, *22* (20), 13243–13268. https://doi.org/10.5194/acp-22-13243-2022.

(14) Wei, J.; Wang, J.; Li, Z.; Kondragunta, S.; Anenberg, S.; Wang, Y.; Zhang, H.; Diner, D.; Hand, J.; Lyapustin, A.; Kahn, R.; Colarco, P.; Da Silva, A.; Ichoku, C. Long-Term Mortality Burden Trends Attributed to Black Carbon and PM2·5 from Wildfire Emissions across the Continental USA from 2000 to 2020: A Deep Learning Modelling Study. *The Lancet Planetary Health* **2023**, *7* (12), e963–e975. https://doi.org/10.1016/S2542-5196(23)00235-8.

(15) Bielen, D. A.; Macpherson, A. J.; Simon, H.; Fann, N. CABOT-O_3 : An Optimization Model for Air Quality Benefit-Cost and Distributional Impacts Analysis. *Environ. Sci. Technol.* **2020**, *54* (21), 13370–13378. https://doi.org/10.1021/acs.est.0c01053.

(16) Macpherson, A. J.; Simon, H.; Langdon, R.; Misenheimer, D. A Mixed Integer Programming Model for National Ambient Air Quality Standards (NAAQS) Attainment Strategy Analysis. *Environmental Modelling & Software* **2017**, *91*, 13–27. https://doi.org/10.1016/j.envsoft.2017.01.008.

(17) Liao, K.-J.; Hou, X. Optimization of Multipollutant Air Quality Management Strategies: A Case Study for Five Cities in the United States. *Journal of the Air & Waste Management Association* **2015**, *65* (6), 732–742. https://doi.org/10.1080/10962247.2015.1014073.

(18) Hochbaum, D. S.; Pathria, A. Analysis of the Greedy Approach in Problems of Maximumk-Coverage. *Naval Research Logistics* **1998**, *45* (6), 615–627. https://doi.org/10.1002/(SICI)1520-6750(199809)45:6%3C615::AID-NAV5%3E3.0.CO;2-5.

(19) Khuller, S.; Moss, A.; Naor, J. (Seffi). The Budgeted Maximum Coverage Problem. *Information processing letters* **1999**, *70* (1), 39–45.

(20) Tessum, C. W.; Paolella, D. A.; Chambliss, S. E.; Apte, J. S.; Hill, J. D.; Marshall, J. D. $PM_{2.5}$ Polluters Disproportionately and Systemically Affect People of Color in the United States. *Sci. Adv.* **2021**, *7* (18), eabf4491. https://doi.org/10.1126/sciadv.abf4491.

(21) Koolik, L. H.; Alvarado, Á.; Budahn, A.; Plummer, L.; Marshall, J. D.; Apte, J. S. PM2.5 Exposure Disparities Persist despite Strict Vehicle Emissions Controls in California. *Science Advances 10* (37), eadn8544. https://doi.org/10.1126/sciadv.adn8544.

(22) Wang, Y.; Apte, J. S.; Hill, J. D.; Ivey, C. E.; Patterson, R. F.; Robinson, A. L.; Tessum, C. W.; Marshall, J. D. Location-Specific Strategies for Eliminating US National Racial-Ethnic PM2.5 Exposure Inequality. *Proc. Natl. Acad. Sci. U.S.A.* **2022**, *119* (44), e2205548119. https://doi.org/10.1073/pnas.2205548119.

(23) Koolik, L. H.; Speizer, S.; Rong, C.; Chambliss, S.; Marshall, J. D.; Morello-Frosch, R.; Tessum, C. W.; Apte, J. S. Methodological Design Choices Can Affect Air Pollution Exposure Disparity Estimates: A Case Study on California's Agricultural Sector. *Environ. Sci. Technol.* **2026**, *60* (6), 4753–4763. https://doi.org/10.1021/acs.est.5c10796.

(24) Industrial Economics. *Evaluating Reduced-Form Tools for Estimating Air Quality Benefits*; 2019. https://www.epa.gov/sites/default/files/2020-09/documents/iec_rft_report_9.15.19.pdf.
